# Naturally occurring mutations in envelope mediate virulence of Usutu virus

**DOI:** 10.1128/mbio.01593-25

**Published:** 2025-09-12

**Authors:** Megan B. Vogt, Jeffrey M. Marano, William J. Hanrahan, Seth A. Hawks, Anne M. Brown, Sheryl Coutermarsh-Ott, James Weger-Lucarelli, Nisha K. Duggal

**Affiliations:** 1 Department of Biomedical Sciences and Pathobiology, Virginia-Maryland College of Veterinary Medicine, Virginia Polytechnic Institute and State University 1757, Blacksburg, Virginia, USA; 2 Department of Biochemistry, Virginia Polytechnic Institute and State University, Blacksburg, Virginia, USA; 3 University Libraries, Virginia Polytechnic Institute and State University 1757, Blacksburg, Virginia, USA; University of Pennsylvania, Philadelphia, Pennsylvania, USA

**Keywords:** Usutu virus, flavivirus, chimeric viruses, viral determinants, virulence

## Abstract

Usutu virus (USUV) is a mosquito-transmitted flavivirus that is closely related to West Nile virus. Recently, USUV emerged in Europe, where it has caused multiple bird die-off events and neuroinvasive disease in humans. Previously, we showed that USUV isolates from Africa cause significantly more severe disease than European isolates in mice. Sequence analysis revealed that the most virulent isolate (Uganda 2012) and the least virulent isolate (Netherlands 2016) differed by 21 amino acids across the viral polyprotein. Here, we sought to identify the viral determinants of and mechanisms for differential virulence. To accomplish this, we used our USUV reverse genetics system and bacteria-free cloning to generate chimeric viruses between Uganda 2012 and Netherlands 2016. *Ifnar1^−/−^* mice infected with a Netherlands 2016 chimera containing all of the structural genes, or just the envelope gene, from Uganda 2012 had significantly higher mortality rates and viremia than those infected with wild-type Netherlands 2016. We were unable to identify a single amino acid in the envelope protein that resulted in significantly increased virulence compared to wild-type Netherlands 2016. These results indicate that multiple mutations in USUV envelope protein contribute to differential virulence between isolates. Through *in vitro* assays, we discovered that envelope mediates replication kinetics, with fusion occurring more slowly for wild-type Netherlands 2016 compared to viruses containing the envelope from Uganda 2012, suggesting a mechanism for envelope-mediated differential virulence of USUV. These studies provide insights into USUV pathogenic mechanisms, which could be used to evaluate the disease potential of related emerging viruses.

**IMPORTANCE:**

Usutu virus (USUV) is currently emerging in Europe, where it has caused numerous mass bird die-off events and neuroinvasive disease in humans. Multiple strains of USUV are circulating throughout Europe, but only some of them have been associated with severe disease in humans. The USUV proteins responsible for and the mechanisms through which they cause severe disease are unknown; however, this information could be invaluable in evaluating disease potential of specific strains and the creation of anti-viral therapies. Here, we swapped genes between USUV strains that cause mild and severe disease and were able to identify a viral protein that mediates virulence. We also discovered that the mild strain of USUV takes significantly longer to complete fusion during viral entry into host cells than the severe strain. This delayed fusion could have impacts on cellular tropism, viral kinetics, susceptibility of the virus to immune responses, and, ultimately, disease severity.

## INTRODUCTION

Usutu virus (USUV; *Flaviviridae*) is a neuroinvasive, mosquito-transmitted flavivirus that is closely related to West Nile virus (WNV). Passerine birds and *Culex* spp. mosquitoes are the reservoir hosts and vectors for USUV, respectively, with humans being dead-end hosts (1–4). Like WNV, USUV can cause neuroinvasive disease in both birds and humans, resulting in mass die-off events in birds and encephalitis or meningitis in humans (5). USUV has followed a similar pattern of global emergence to WNV. Originally isolated in South Africa in 1959, USUV has been spreading throughout Europe since the late 1990s (6–9), resulting in several mass die-offs of the Eurasian blackbird (*Turdus merula*) and neuroinvasive disease in humans (5, 7, 10–18).

USUV isolates have been grouped into eight lineages (Africa 1–3 and Europe 1–5) based on genetic similarity of the viral polymerase gene (19). Previously, we compared virulence in the *Ifnar1^−/−^* mouse model of contemporary USUV isolates from multiple lineages: Africa 2 (Senegal 2003 and Spain 2009), Africa 3 (Netherlands [NE] 2016) and Europe 5 (Uganda [UG] 2012) (20). While all isolates grew similarly *in vitro*, there were significant differences in mortality and viremia (serum virus concentration), with UG2012 and NE2016 exhibiting the most and least virulence, respectively. These two isolates differ by 21 amino acids across the entire polyprotein; specific viral proteins that differ between the two isolates include capsid, envelope, non-structural (NS)1, NS2a, NS3, NS4b, and NS5.

Multiple flavivirus proteins have been implicated in virulence. The envelope protein, a structural protein found on the outer surface of the virion, binds receptors on host cells, mediates fusion of the virus to endosomal membranes, and is a target of host antibody responses. Mutations in envelope protein can significantly impact the virulence of WNV, Murray Valley encephalitis virus, tick-borne encephalitis virus, Japanese encephalitis virus, and others (21–28). Extracellular NS1 can modulate vascular leakage in tissue-specific and viral-specific manners in dengue, Zika, Japanese encephalitis, and yellow fever viruses (29, 30). Additionally, mutations in either NS3 (viral protease and helicase) or NS5 (viral polymerase and methyltransferase) can significantly impact the virulence of yellow fever, Japanese encephalitis, dengue, Kunjin, and tick-borne encephalitis viruses (31–33). For USUV, viral determinants of disease are unknown.

Here, we investigated which viral proteins are responsible for the differential virulence observed between the USUV isolates UG2012 and NE2016. Using bacteria-free cloning techniques, we created chimeric viruses in which multiple genes and single genes were swapped between UG2012 and NE2016. Virulence of these newly created chimeric viruses was evaluated in the *Ifnar1*^−/−^ mouse model. We discovered that the envelope protein mediates USUV virulence, and multiple mutations are required for envelope-mediated virulence. Finally, we demonstrated that viruses containing envelope protein from UG2012 have faster fusion rates, highlighting a possible mechanism for the differential virulence observed between UG2012 and NE2016. These studies are crucial to understanding the virulence of emerging USUV in Europe and for understanding how the evolution of flaviviruses impacts virulence in mammalian hosts.

## MATERIALS AND METHODS

Additional method details can be found in Supplemental methods.

### Cells and mice used

Vero cells (ATCC; *Cercopithecus aethiops*) were grown in Dulbecco’s modified Eagle medium (DMEM; Corning) supplemented with 5% fetal bovine serum (FBS; Avantor) and 1% penicillin/streptomycin mix (Gibco) at 37°C in a humidified incubator. BHK-21 (ATCC; *Mesocricetus auratus*) cells were grown in DMEM supplemented with 10% FBS and 1% penicillin/streptomycin mix. *Ifnar1*^−/−^ mice were originally purchased from The Jackson Laboratory (Bar Harbor, ME, USA). Additional mice were bred at Virginia Tech. All mice used in studies were 6-11 weeks old. Both male and female mice were used in these studies and were spread evenly across the study groups.

### Generation of infectious clones, chimeric viruses, and point mutants

Infectious clones, chimeric viruses, and point mutants were made using bacteria-free cloning. Briefly, viruses were designed in SnapGene (v.6.0.2 through v.8.0.01). Viral genome fragments were PCR amplified out of previously generated infectious clones or synthesized DNA (Twist Biosciences). Genome fragments were assembled and amplified using the OriCiro kit following the manufacturer’s instructions. Additional amplification occurred via rolling circle amplification using the Equiphi polymerase (ThermoFisher). Viruses were rescued in Vero or BHK-1 cells. Viruses were sequence-verified and validated via *in vitro* growth curves.

### Mouse experiments

*Ifnar1^−/−^* mice were infected in groups of eight with 1,000 plaque-forming units (PFUs) in a 40 µL dose via subcutaneous injection in the rear footpad (20). Mice were monitored daily to assess for signs of disease. Blood was collected on days 3, 4, and 5 post-inoculation and immediately prior to euthanasia via submandibular bleed. Serum was separated from whole blood using serum separator tubes (Sarstedt or BD Biosciences) via the manufacturer’s instructions. Serum samples were stored at −80°C until titration via plaque assay. Mice were euthanized if they lost more than 15% of their starting weight, appeared moribund (hunched back, ruffled fur, refusal to move), or at the termination of the study (day 13). Upon euthanasia, brains were collected from each mouse. Half of each brain was stored at −80°C until titration via plaque assay, while the other half was fixed in 10% buffered formalin for histology.

### Structure modeling methods

Structural models of USUV envelope proteins of NE2016 and UG2012 were generated using Robetta Comparative Modeling (34). NetNGlyc was used to identify glycosylation sites on both the Uganda and Netherlands strains (35).

### Time-to-fusion assay

Virus (1,000 PFUs) was allowed to adsorb for 1 h at 4°C on to Vero cells at 90% confluency. Unbound virus was removed by washing with ice-cold PBS. Then, the media were added, and the cells were incubated at 37°C for 1 h. At 15-min intervals, the media were removed from specified wells and replaced with media containing 200 mM ammonium chloride (NH_4_Cl) solution. At the conclusion of the incubation, NH_4_Cl was removed by washing with PBS. From here, plates were treated as in plaque assay.

### Plaque reduction neutralization titer (PRNT) assay

Neutralizing antibody titers in mouse serum were quantified using a PRNT assay as described in reference 36. Briefly, serum collected from infected mice upon euthanasia was heat-inactivated, and a twofold dilution series was made. An equal volume of USUV was added to serum dilutions. Virus diluted 1:2 in media served as a no-antibody control. Virus and antibody were incubated at 37°C for 1 hour. From here on, plates were treated as in plaque assay.

### Phylogenetic analyses

USUV sequences for phylogenetic analysis were selected from GenBank. Isolation dates for sequenced viruses ranged from 1959 to 2021. Sequences were aligned via Clustal Omega and trimmed to include only the coding region (37). Identical sequences were identified and removed using sRNAtoolbox Helper Tools (38). Phylogenetic trees of the remaining 74 sequences were constructed using PhyML (v.3.0) with 1,000 bootstraps (39). The resulting tree was visualized using FigTree (v.1.4.4). USUV lineages were determined by referencing previous phylogenetic analyses (40).

### Statistical analyses

Statistical analyses were performed using GraphPad Prism (v.10.3.1). Data from *in vitro* multi-step growth curves, mouse weights, mouse viremia, and fusion timing assay were analyzed using two-way ANOVA followed by multiple comparisons *t*-tests using Dunnett’s adjustment. Survival data were analyzed using the Mantel-Cox test and adjusted for multiple comparisons via the Holm-Sidak method. Brain titers and single-step growth curves were analyzed using one-way ANOVA followed by multiple comparisons *t*-tests using Dunnett’s adjustment. Histology scores were analyzed via the Mann-Whitney test and adjusted for multiple comparisons using the Holm-Sidak method. Correlation analyses were performed using the Spearman correlation coefficient.

## RESULTS

### UG2012ic is more virulent than NE2016ic in *Ifnar1^−/−^* mice

**Fig 1:**
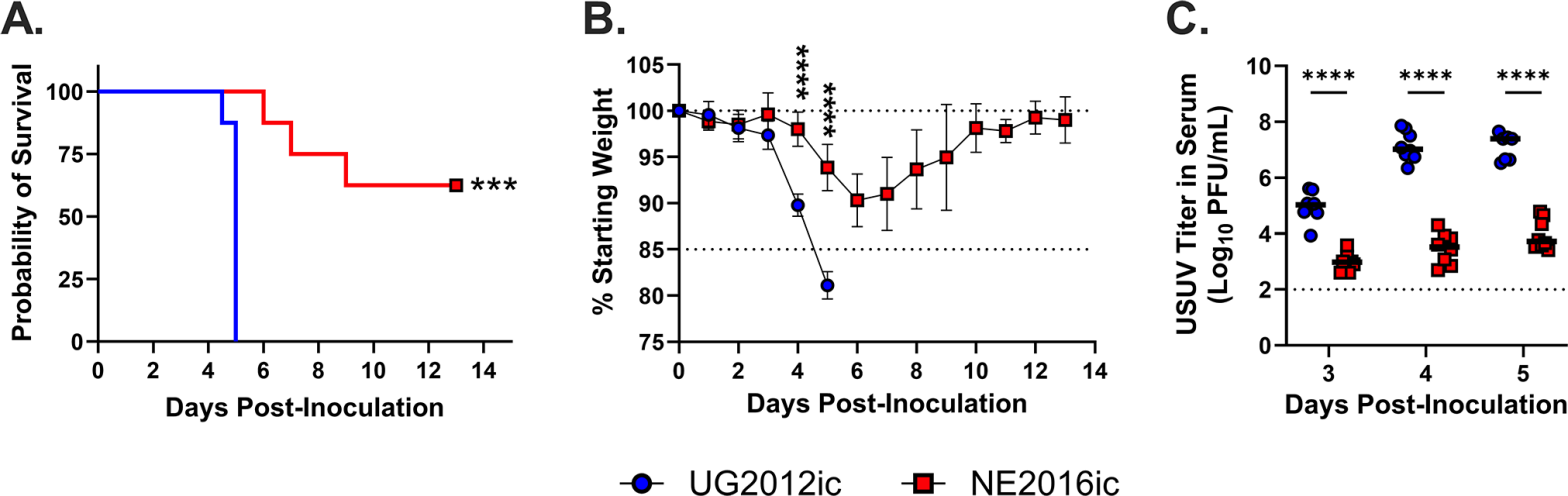
UG2012ic is more virulent in *Ifnar1^−/−^* mice than NE2016ic. Adult *Ifnar1*^−/−^ mice were infected via subcutaneous footpad inoculation with 1,000 PFUs of either UG2012ic (*N* = 8; blue circles) or NE2016ic (*N* = 8; red squares). Survival (**A**), change in weight (**B**), and viremia (**C**) were assessed at the indicated time points. Survival data were analyzed via the Mantel-Cox test. Weight data and viremia data were analyzed via two-way ANOVA followed by multiple comparisons *t*-test using the Sidak correction. Weight curves of individual mice can be found in Fig. S2. ns, not significant; ***P < 0.001 and ****p < 0.0001.

We previously created infectious clones (ic) of the UG2012 (GenBank MN813491.1) and NE2016 (GenBank MN813490.1) isolates (41). The amino acid sequences of these clones each differ by one amino acid in envelope protein from the isolates: NE2016ic, E-K292E; and UG2012, E-I213V. Further analysis of the isolates revealed polymorphisms at those sites with a mixed population of the original sequence and the clone sequence. The clones performed similarly in *in vitro* and *in vivo* models compared to parental isolates (41). Here, we adapted the infectious clones (ic) for both UG2012 and NE2016 to use a cell-free method of DNA amplification reliant on replication cycle reaction, which yields supercoiled, plasmid, icDNA that is easier to manipulate and transfect into eukaryotic cells than that generated from traditional bacteria-free cloning methods (42–45). Following rescue, sequence verification, and validation of growth kinetics (Fig. S1), we assessed the pathogenicity of UG2012ic and NE2016ic in adult *Ifnar1*^−/−^ mice, a commonly used mouse model of flavivirus infection (46). We observed significantly lower survival rates in animals infected with UG2012ic (0% survival) compared to NE2016ic (63% survival; *P* = 0.0001; Fig. 1A; Table S1). To assess the morbidity of mice, we measured weight daily (Fig. 1B; Fig. S2). Overall, the pattern of weight loss between UG2012ic and NE2016ic differed (*P* < 0.0001). Specifically, animals infected with UG2012ic lost significantly more weight than those infected with NE2016ic on day 4 post-inoculation (*P* < 0.0001) and day 5 post-inoculation (*P* < 0.0001). Finally, we assessed viremia in the infected animals (Fig. 1C). Mice infected with UG2012ic had significantly higher titers than those infected with NE2016ic at day 3 (*P* < 0.0001), day 4 (*P* < 0.0001), and day 5 post-inoculation (*P* < 0.0001). Taken together, these data indicate that UG2012ic is more virulent than NE2016ic in *Ifnar1*^−/−^ mice. These results are consistent with our previous study in which differential virulence was observed between UG2012 and NE2016 isolates in *Ifnar1*^−/−^ mice (20).

### USUV structural proteins mediate virulence

**Fig 2:**
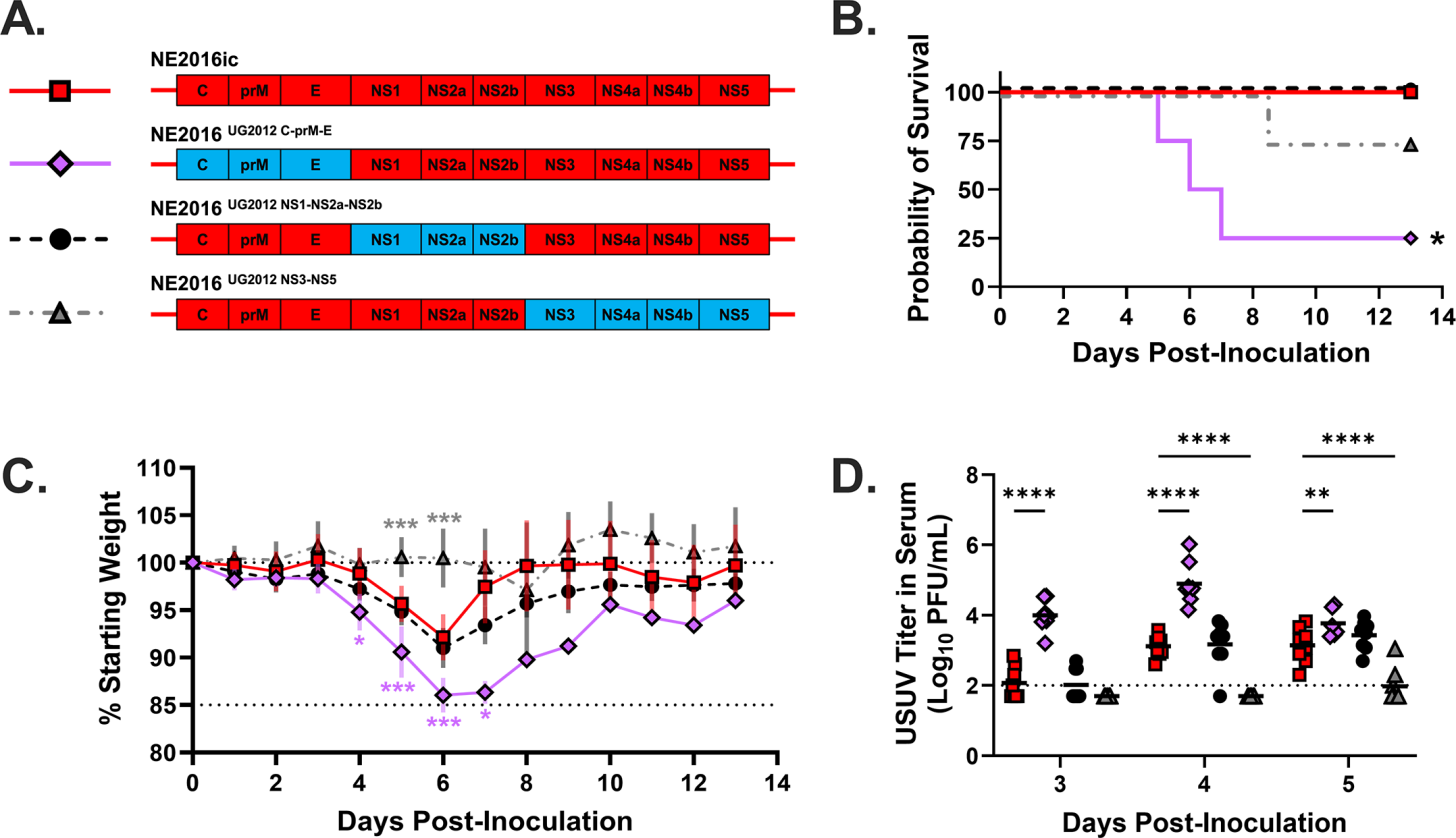
Structural proteins mediate USUV virulence. Multi-gene chimeras containing UG2012 (blue) with the NE2016 backbone (red) were created (**A**). Adult *Ifnar1*^−/−^ mice were infected via subcutaneous footpad inoculation with 1,000 PFUs of NE2016ic (*N* = 8; red squares), NE2016^UG2012 C-PrM-E^ (*N* = 8; purple diamonds), NE2016^UG2012 NS1-NS2a-NS2b^ (*N* = 8; black circles), or NE2016^UG2012 NS3-NS5^ (*N* = 8; gray triangles). Survival (**B**), change in weight (**C**), and viremia (**D**) were assessed at the indicated time points. For viremia, points below the limit of detection represent samples in which the virus was not detectable. Survival data were analyzed via the Mantel-Cox test, and *P* values were adjusted for multiple comparisons using the Holm-Sidak correction. Weight data and viremia data were analyzed via two-way ANOVA followed by multiple comparisons *t*-test using the Dunnett correction. For all analyses, the chimeras were compared to NE2016ic. Weight curves of individual mice can be found in Fig. S3. *P < 0.05; **P < 0.005; and ****P < 0.0001.

** **Next, we sought to identify the viral determinants of the differential virulence phenotype that we observed in *Ifnar1^−/−^* mice. NE2016ic and UG2012ic have 97.4% nucleotide similarity and 99.4% amino acid similarity. There are 21 amino acid differences between NE2016ic and UG2012ic polyproteins (Table 1). To narrow down the specific proteins for differential virulence, we generated chimeric viruses in which multi-gene segments of the UG2012ic genome were inserted into NE2016ic. The chimeras made were NE2016^UG2012 C-prM-E^, NE2016^UG2012 NS1-NS2a-NS2b^, and NE2016^UG2012 NS3-NS5^ (Fig. 2A). 

**TABLE 1:** Amino acid differences between UG2012ic and NE2016ic

Protein (abbreviation)	Amino acid # (within protein)	UG2012ic	NE2016ic
Capsid (C)	105	G	S
	112	V	L
Envelope (E)	345 (52)	N	S
	381 (88)	D	N
	472 (179)	K	E
	524 (231)	S	L
	531 (238)	I	T
	637 (344)	S	T
NS1	843	H	Y
	891	R	K
NS2A	1,268	F	L
	1,287	A	V
	1,322	I	V
	1,334	A	V
NS3	1,549	L	F
	1,602	I	V
	1,983	N	S
	2,059	I	V
NS4B	2,460	L	F
NS5	2,803	A	S
	3,060	K	R

To determine which of these gene portions impacts USUV virulence, we infected *Ifnar1*^−/−^ mice with the aforementioned chimeras and compared morbidity, mortality, and viral titers to mice infected with NE2016ic (Fig. 2). We observed significantly lower survival in animals infected with NE2016^UG2012 C-prM-E^ chimera compared to those infected with NE2016ic (*P* = 0.025; Fig. 2B; Table S1). Animals infected with NE2016^UG2012 C-prM-E^ experienced significantly more weight loss than those infected with NE2016ic on day 4 (*P* = 0.011), day 5 (*P* = 0.0023), day 6 (*P* = 0.0005), and day 7 (*P* = 0.016) post-inoculation (Fig. 2C; Fig. S3). Animals infected with the NE2016^UG2012 NS3-NS5^ chimera lost significantly less weight than the NE2016ic group on day 5 (*P* = 0.0007) and day 6 (*P* = 0.0001) post-inoculation. Viremia was assessed on days 3, 4, and 5 post-inoculation (Fig. 2D). The mean viremia of animals infected with NE2016^UG2012 C-prM-E^ was significantly higher than those infected with NE2016ic on day 3 (*P* < 0.0001), day 4 (*P* < 0.0001), and day 5 (*P* < 0.0001) post-inoculation. Viremia was only detected in the NE2016^UG2012 NS3-NS5^ group on day 5 post-inoculation. The mean titer at this time was significantly lower than that of the NE2016ic group (*P* < 0.0001). Taken together, these results indicate that the structural proteins mediate USUV virulence, as indicated by lower survival rates and higher viremia titers in animals infected with the NE2016^UG2012 C-prM-E^ chimera compared to NE2016ic.

### USUV envelope protein mediates differential virulence

**Fig 3:**
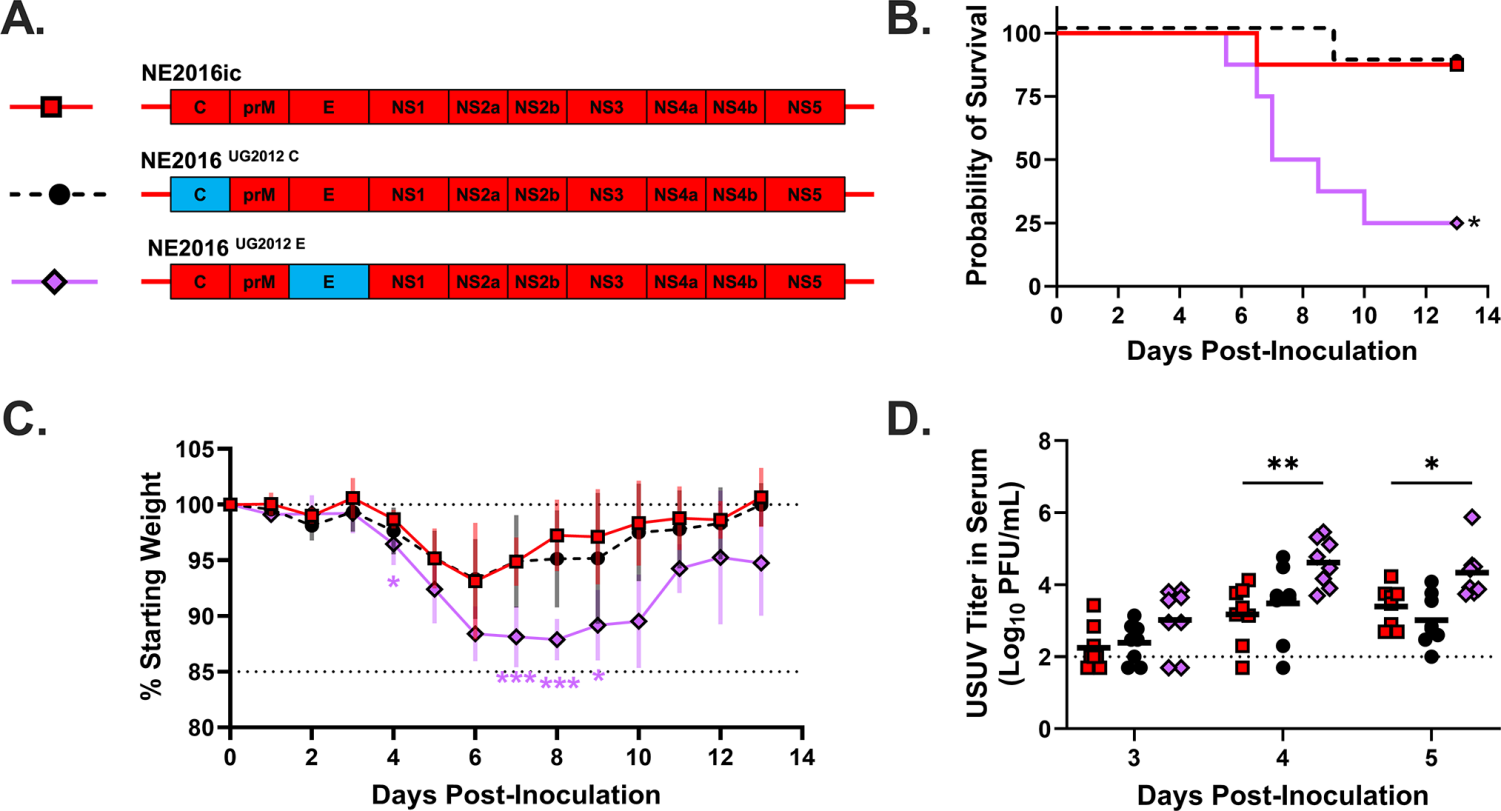
USUV envelope protein mediates virulence. Single gene chimeras containing UG2012 (blue) with the NE2016 backbone (red) were created (**A**). Adult *Ifnar1*^−/−^ mice were infected via subcutaneous footpad inoculation with 1,000 PFUs of NE2016ic (*N* = 8; red squares), NE2016^UG2012 Capsid^ (*N* = 8; black circles), or NE2016^UG2012 Envelope^ (*N* = 8; purple diamonds). Survival (**B**), change in weight (**C**), and viremia (**D**) were assessed at the indicated time points. For viremia, points below the limit of detection represent samples in which the virus was not detectable. Survival data were analyzed via the Mantel-Cox test, and *P* values were adjusted for multiple comparisons using the Holm-Sidak correction. Weight data and viremia data were analyzed via two-way ANOVA followed by multiple comparisons *t*-test using the Dunnett correction. For all analyses, the chimeras were compared to NE2016ic. Weight curves of individual mice can be found in Fig. S4. *P < 0.05 and **P < 0.01.

Next, we sought to determine which of the structural proteins mediate differential virulence. To do this, we generated chimeras in which the capsid (C) or envelope (E) genes from UG2012ic were inserted into the NE2016ic background (Fig. 3A). We did not make a chimera swapping prM, because no amino acid differences exist between UG2012ic and NE2016ic in prM. To assess virulence of these chimeric viruses, we infected adult *Ifnar1*^−/−^ mice with them and compared morbidity, mortality, and viral load to mice infected with NE2016ic. We observed a significantly lower survival rate in animals infected with NE2016^UG2012 E^ compared to those infected with NE2016ic (*P* = 0.019; Fig. 3B; Table S1). The survival rate of the NE2016^UG2012 C^ group was the same as the NE2016ic group. There were no significant differences in weight loss between mice infected with NE2016ic versus NE2016^UG2012 C^ (Fig. 3C, Fig. S4). In contrast, animals infected with NE2016^UG2012 E^ lost significantly more weight than those infected with NE2016ic at day 4 (*P* = 0.023), day 7 (*P* = 0.0014), day 8 (*P* = 0.0003), and day 9 (*P* = 0.038) post-inoculation. Viremia was assessed on days 3, 4, and 5 post-inoculation (Fig. 3D). The mean titer of the NE2016^UG2012 C^ group did not significantly differ from the NE2016ic group at any time point. The mean titer of the NE2016^UG2012 E^ group was significantly higher than that of the NE2016ic group at day 4 (*P* = 0.0035) and day 5 (*P* = 0.021) post-inoculation. Taken together, these data indicate that USUV envelope protein plays a key role in mediating virulence, as indicated by lower survival rates and higher viremia in animals infected with NE2016^UG2012 E^ compared to those in animals infected with NE2016ic.

### Envelope-mediated differential virulence requires multiple amino acids

**Fig 4:**
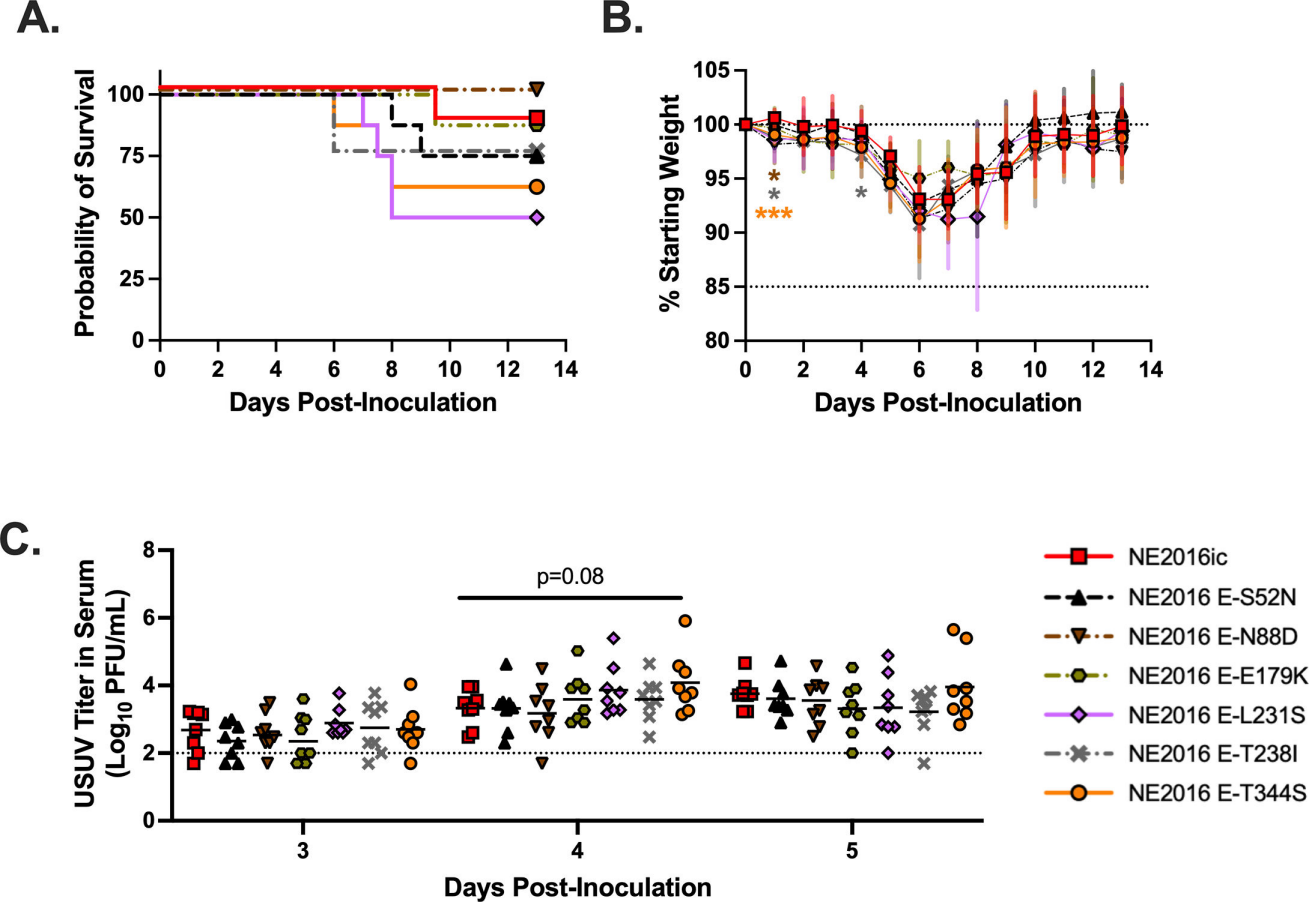
USUV envelope virulence is not mediated by a single amino acid. Adult *Ifnar1*^−/−^ mice were infected via subcutaneous footpad inoculation with 1,000 PFUs of NE2016ic (*N* = 8; red squares), NE2016 E-S52N (*N* = 8; black triangles), NE2016 E-N88D (*N* = 8; brown inverted triangles), NE2016 E-E179K (*N* = 8; yellow-ochre hexagons), NE2016 E-L231S (*N* = 8; purple diamonds), NE2016 E-T238I (*N* = 8; gray Xs), or NE2016 E-T344S (*N* = 8; orange circles). Survival (**A**), change in weight (**B**), and viremia (**C**) were assessed at the indicated time points. For viremia, points below the limit of detection represent samples in which the virus was not detectable. Survival data were analyzed via the Mantel-Cox test, and *P* values were adjusted for multiple comparisons using the Holm-Sidak correction. Weight data and viremia data were analyzed via two-way ANOVA followed by multiple comparisons *t*-test using the Dunnett correction. For all analyses, the mutants were compared to NE2016ic. Weight curves of individual mice can be found in Fig. S5. *P < 0.05.

Next, we sought to identify which amino acid(s) in USUV envelope protein mediate virulence. The envelope proteins in UG2012ic and NE2016ic differ by six amino acids (Table 1). We generated six point mutants of NE2016ic envelope, each with a different amino acid change that matched UG2012ic at that position: E-S52N, E-N88D, E-E179K, E-L231S, E-T238I, and E-T344S. To assess virulence of these mutants, we infected adult *Ifnar1*^−/−^ mice and assessed morbidity, mortality, and viremia (Fig. 4). Using spot sequencing on serum samples, we confirmed that the viruses did not revert to wild-type during infection. None of the groups had survival rates that significantly differed from NE2016ic (88%; Fig. 4A; Table S1). Overall, the weight loss curves are similar between groups. Significant differences occur between NE2016ic and E-N88D (*P* = 0.023), E-T238I (*P* = 0.029), and E-T344S (*P* = 0.0075) on day 1 and NE2016ic and E-T344S on day 4 (Fig. 4B; Table S5). However, given the small magnitude of the differences in weight loss, it is unclear whether these are biologically significant. Viremia was assessed on days 3, 4, and 5 post-inoculation (Fig. 4C). Viral titers from animals infected with any of the point mutants were not significantly different from those in the NE2016ic group at any time point; however, on day 4 post-inoculation, there was a trend toward higher viremia in animals infected with the E-T344S mutant compared to NE2016ic (*P* = 0.08). Taken together, these results show that a single amino acid change in the envelope protein of NE2016 is not sufficient to augment virulence as indicated by the lack of significant changes in mortality and viremia between mice infected with any of the point mutants versus wild-type NE2016ic.

### USUV and pathologies were detected in the brain of animals that succumbed to infection

**Fig 5:**
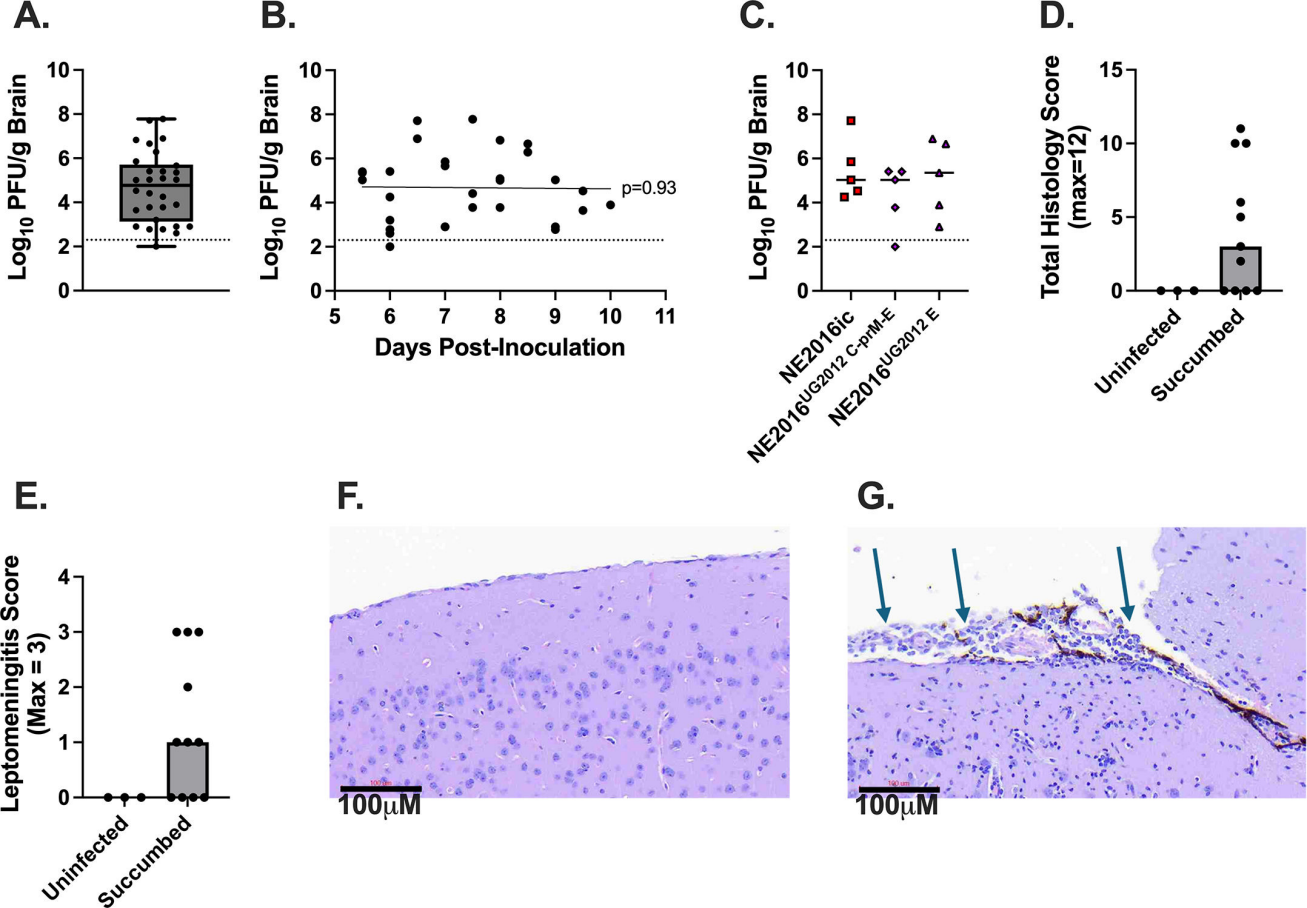
Succumbed animals show signs of neuroinvasive disease. Brains from mice that succumbed to infection (*n* = 30) from NE2016ic, any chimeric virus, or any point mutant were assessed for signs of neuroinvasive disease. (**A**) Brain titers for all succumbed mice, regardless of virus, are displayed via box and whisker plot, with the line in the middle of the box representing the median, the box representing the interquartile range, and the whiskers representing the range. (**B**) Brain titers are plotted by day of euthanasia, with the line showing results of a linear regression analysis and the *P* value showing that the slope of the line is not different from 0. (**C**) Brain titers from animals that succumbed from infection with NE2016ic (*N* = 5), NE2016^UG2012 C-prM-E^ (*N* = 5), or NE2016^UG2012 E^ (*N* = 5). Lines represent median values. Data were analyzed using multiple *t*-tests, using the Dunnett’s multiple comparisons adjustment. For (**A–C**), points plotted underneath the limit of detection represent samples where the virus was undetectable. Total pathology scores (**D**) as well as scores for leptomeningitis (**E**) are graphed for succumbed (*N* = 11) and uninfected (*N* = 3) mice with points representing individual mice and gray boxes representing the median. Representative images of uninflamed (NE2016-infected) (**F**) and inflamed (NE2016^UG2012 E^-infected) (**G**) leptomeninges are shown. Arrows in (**G**) highlight inflammatory cells.

Since severe USUV disease in humans results in neurological complications, we investigated whether severe USUV disease in mice was also accompanied by signs of neuroinvasive disease. To that end, we evaluated viral load and pathology of brains from the mice in Fig. 1 through 4 that succumbed after infection with NE2016 backbone viruses (*N* = 30). USUV was detected in the brains of 97% of animals that succumbed to infection. The median USUV titer in the brain was 4.8 log_10_ PFU/g, with titers from individual animals ranging from 2.6 to 7.8 log_10_ PFU/g (Fig. 5A). Because mice succumbed at different days post-infection, we evaluated whether viral titer was associated with day of euthanasia (Fig. 5B). Linear regression (slope = −0.02; *P* = 0.93) and correlation (Spearman *r* = −0.007; *P* = 0.97) analyses revealed that there was no relationship between brain titer and day of euthanasia. Median brain titers were not different between animals that succumbed to NE2016ic versus NE2016^UG2012 C-prM-E^ (*P* = 0.72) or NE2016^UG2012 E^ (*P* = 0.99) (Fig. 5C).

Next, we evaluated brain pathology on a subset of mice that succumbed to USUV infection (*n* = 11). Brain sections were evaluated based on evidence of inflammation, necrosis, vascular lesion, and leptomeningitis. Most brain samples exhibited inflammation and/or necrosis. Many samples exhibited some degree of cell death characterized by individual or aggregates of shrunken, hypereosinophilic (intensely pink staining) cells with fragmented nuclei. Parenchymal inflammation most consistently appeared as gliosis, or expansion of glial cells throughout the sections. Occasionally, gitter cells (macrophages in the brain), lymphocytes, plasma cells, or neutrophils were also observed. Similarly, perivascular inflammation and inflammation within the leptomeninges (leptomeningitis) were dominated by mononuclear cells but sporadically included neutrophils. These changes were graded semi-quantitatively to produce a total histologic score. The median total histology score for succumbed animals was 3 (maximum score = 12), with scores ranging from 0 to 11 (Fig. 5D). Additionally, the median leptomeningitis score for succumbed animals was 1 (maximum score = 3), with scores ranging from 0 to 3 (Fig. 5E). Inflamed meninges can be visualized in Fig. 5G, a brain slice of an animal infected with NE2016^UG2012 E^ that had a leptomeningitis score of 3. As a comparison, normal meninges can be visualized in Fig. 5F, a brain slice of an animal infected with NE2016ic that had a leptomeningitis score of 0.

Taken together, these results show that severe USUV infection is associated with neuroinvasive disease in mice, as evidenced by the presence of USUV and pathologies, including leptomeningitis, in brains of mice that succumbed to USUV infection.

### USUV envelope protein impacts viral fusion kinetics but does not impact neutralizing antibody titers

**Fig 6:**
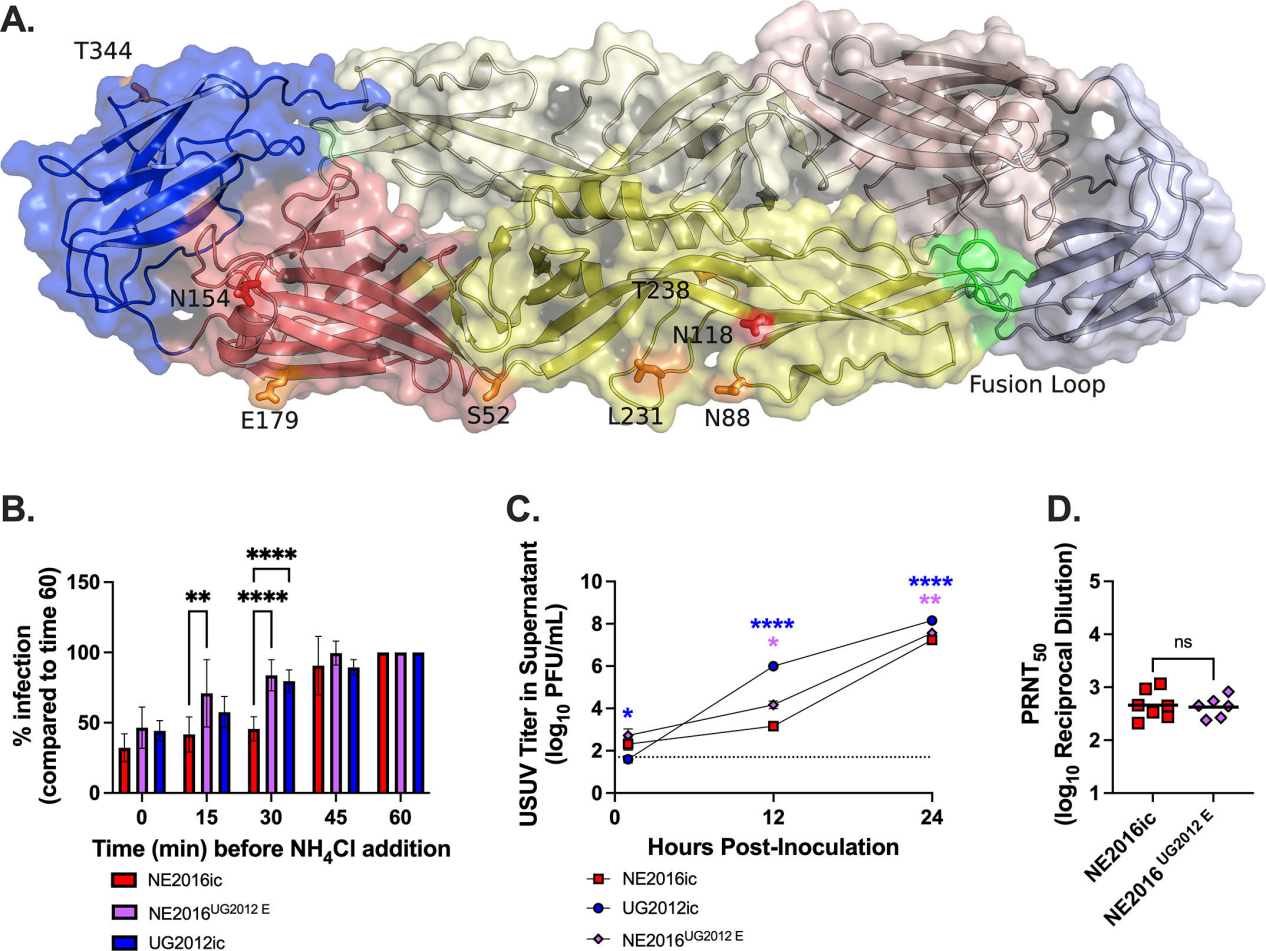
USUV envelope protein impacts speed of viral entry. (**A**) Ribbon structure of NE2016 envelope in dimer configuration overlaid on the surface map. The fusion loop on each monomer is highlighted in green. Six amino acid mutations are labeled and highlighted in orange. N-linked glycosylation sites are highlighted in red. (**B**) Viral entry speeds of NE2016, NE2016^UG2012 E^, and UG2012 were assessed via time to fusion assay in Vero cells. Data are the mean and standard deviation of at least three independent replicates. Data were analyzed via one-way ANOVA with multiple comparisons *t*-test using the Dunnett’s correction. (**C**) Single-step growth curve in Vero cells with NE2016 (red), NE2016^UG2012 E^ (lavender), and UG2012 (blue) at MOI of 10. Data are the mean and standard deviation of three technical replicates of a representative biological replicate. Additional biological replicates can be found in Fig. S6. Data were analyzed via two-way ANOVA with multiple comparisons *t*-test using the Dunnett’s correction. Asterisks are colored to show which virus was significantly different from NE2016ic. (**D**) Neutralizing antibody titers in terminal serum samples for mice infected with NE2016ic (red squares) or NE2016^UG2012 E^ (purple diamonds) were quantified using PRNT assay, using the same viruses with which mice were originally infected (i.e., NE2016ic or NE2016^UG2012 E^). Mice that were still viremic at the time of euthanasia were excluded. PRNT_50_ titers are expressed as the log_10_ reciprocal dilution. Horizontal lines represent the mean of each group. Data were analyzed via student’s *t*-test. min, minute; NH_4_Cl, ammonium chloride; **P < 0.01; ****P < 0.0001; n.s., not significant.

To gain insights into the mechanism through which USUV envelope mediates differential virulence, we modeled and analyzed the structures of envelope protein from both NE2016 and UG2012. With only six amino acid substitutions distinguishing the NE2016 and UG2012 envelope proteins, the secondary and tertiary structural differences between the two strains are minimal (Fig. 6A). These substitutions are not predicted to alter hydrophobicity of the protein or impact the two previously described N-linked glycosylation sites at residues E-118 and E-154 (47). Two substitutions (E-S52N and E-N88D) are predicted to affect other putative N-linked glycosylation sites, potentially enabling a glycosylation site at residue E-52 in UG2012 or at residue E-88 in NE2016. These glycosylation sites have yet to be experimentally validated and are not predicted to cause any observable secondary or tertiary structural changes in the proteins.

** **Because four out of six envelope mutations are located in DII, which contains the fusion domain, we sought to investigate whether the USUV envelope impacts fusion kinetics by performing a time-to-fusion assay in Vero cells with NE2016ic, UG2012ic, and NE2016^UG2012 E^ using ammonium chloride (NH_4_Cl) as a fusion inhibitor (48, 49). When we compared the results of individual viruses with each other, we found that NE2016^UG2012 E^-infected cells treated with NH_4_Cl at 15 min post adsorption had a significantly higher infection rate than NE2016-infected cells treated at the same time point (Fig. 6B). Additionally, both NE2016^UG2012 E^ and UG2012ic infected cells treated with NH_4_Cl at 30 minutes post adsorption had significantly higher infection rates than NE2016-infected cells (Fig. 6B). These data indicate that fusion begins earlier in cells infected with UG2012ic and NE2016^UG2012 E^, both of which contain the envelope protein from UG2012, than in cells infected with NE2016ic.

** **Next, we investigated whether fusion kinetics impacted replication kinetics early in infection. To test this, we performed single-step growth curves of NE2016, UG2012, and NE2016^UG2012 E^ at an MOI of 10 in Vero cells. At twelve hours post-inoculation, we observed significantly higher viral titers in the supernatant of cells infected with UG2012ic (*P* < 0.0001) or NE2016^UG2012 E^ (*P* = 0.01) compared to cells infected with NE2016ic (Fig. 6C). These results indicate that USUV envelope protein impacts single-cycle replication kinetics *in vitro*.

Envelope protein is also the main target of neutralizing antibodies, and even a single mutation in envelope protein can impact the production and function of neutralizing antibodies (21, 50, 51). Therefore, we investigated whether the virulence following NE2016^UG2012 E^ infection was a result of lower neutralizing antibody titers than those generated during NE2016ic infection. We quantified neutralizing antibodies in serum collected at euthanasia from NE2016ic and NE2016^UG2012 E^-infected mice. The mean PRNT_50_ titers were similar between NE2016ic and NE2016^UG2012 E^-infected mice (Fig. 6D). These results indicate that the differential virulence between NE2016ic and NE2016^UG2012 E^ is not due to differences in neutralizing antibody titers.

### NE2016 envelope mutations are found in nearly all Africa 3 lineage viruses

**Fig 7:**
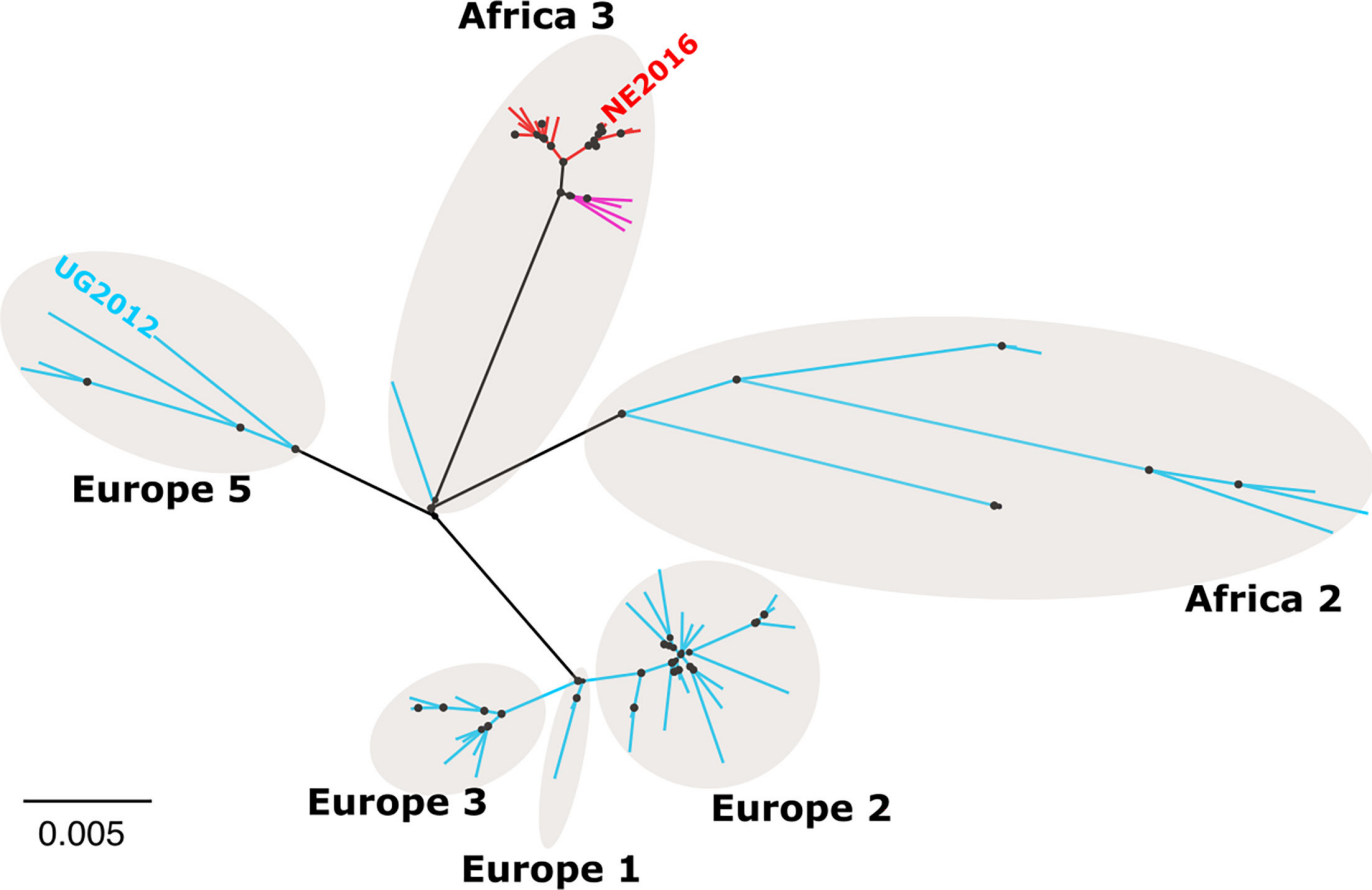
Envelope mutations found in NE2016 are prevalent in the Africa 3 lineage of USUV. A phylogenetic tree made from 74 USUV sequences. Different lineages are highlighted by tan circles and labeled. Tips in teal represent viruses that match UG2012 at all six residues in envelope protein. Tips in red represent viruses that match NE2016 at all six residues in envelope protein. Tips in pink represent viruses that match NE2016 at five out of six residues in envelope protein. Circles at the nodes are sized to represent bootstrap values.

Finally, we sought to identify whether the six amino acid differences in the envelope protein of NE2016 compared to that of UG2012 were unique to NE2016. We performed phylogenetic analysis on over 70 full genome sequences publicly available in GenBank (Table S2) and found multiple USUV isolates that matched NE2016 at all six envelope residues evaluated here. All of these sequences cluster together (Fig. 7) and were identified to be within the Africa 3 lineage of USUV. A small subset of Africa 3 lineage isolates matches NE2016 at five out of six envelope residues; the residue that differs in this subset is residue E-231, and it is a serine (matching UG2012) as opposed to a leucine (matching NE2016). All other sequences that were analyzed in Africa 2, Europe 1, Europe 2, Europe 3, and Europe 5 lineages matched UG2012 at the six envelope residues. This suggests that the decreased virulence of NE2016 is likely a shared feature across many isolates of the Africa 3 lineage.

## DISCUSSION

 USUV is a flavivirus that can cause neuroinvasive disease in humans. Previously, we showed that two USUV strains, NE2016 and UG2012, have different virulence phenotypes in *Ifnar1*^−/−^ mice (20). Here, we sought to determine the viral determinants of this differential virulence phenotype. We discovered that envelope protein mediates USUV virulence, potentially through differential fusion kinetics. Additionally, we determined that USUV can cause neuroinvasive disease in a mouse model.

We used the *Ifnar1*^−/−^ mouse model for these studies, which is a common mouse model for studying flavivirus pathogenesis and has been used by us and others to study USUV infection (46). Wild-type mice have an inherent resistance to several flaviviruses, including USUV, and antagonism of the interferon response is necessary to infect mice with USUV (52, 53). USUV infection in *Ifnar1*^−/−^ mice tends to be lethal, with the exception of infection with NE2016. Recently, we have described a non-lethal mouse model of USUV infection in which wild-type animals were transiently immunosuppressed via treatment with an antibody that temporarily blocks the type I interferon receptors; however, NE2016 causes little to no viremia in this model (41). It should be noted, however, that humans who develop severe USUV disease tend to be older, immunosuppressed, and/or have comorbidities, all of which can have significant impacts on anti-viral immune responses (5). Additionally, anti-interferon antibodies have been detected in individuals experiencing severe disease following infection with USUV or other flaviviruses (54, 55). This evidence supports the use of an immunocompromised mouse model to study USUV infection.

 USUV can cause significant neurological complications, including meningitis and encephalitis, in humans. In the study presented here, we observed clear signs of brain pathology, including leptomeningitis, indicating that the *Ifnar1*^−/−^ mouse model can be used to study USUV neuroinvasive disease. To our knowledge, ours is the first study to visualize USUV-associated meningitis in mice that are infected peripherally. At this point, it is unknown whether USUV neuroinvasion or meningitis occurs in mice without severe clinical disease. Because our studies were designed to assess survival and not neuroinvasion, we were unable to determine whether USUV envelope protein mediates neuroinvasive disease. Future studies will incorporate planned euthanasia between days 6 and 11 post-infection to determine whether USUV can be detected in the brains of mice that experience milder USUV disease.

Using a chimeric virus approach, we identified envelope protein as a key mediator of USUV virulence; however, envelope is likely not the only viral determinant of severe USUV disease. In our studies, the mortality rates and viremia observed in mice infected with either NE2016^UG2012 C-prM-E^ or NE2016^UG2012 E^ were significantly higher than those of mice infected with NE2016ic, but neither chimera completely recapitulates the disease phenotype of UG2012ic. Epistatic interactions between envelope protein and another viral protein may be necessary to fully recapitulate the UG2012ic phenotype. The envelope proteins of NE2016 and UG2012 differ by six amino acids. Using point mutants, we were unable to identify a single amino acid responsible for differential virulence between NE2016 and UG2012; however, we did identify two residues, E-231 and E-344, which trended toward having significant impacts on survival and viremia, respectively. Future studies will utilize double mutants to determine the minimal changes necessary in USUV envelope protein to impact virulence.

 Since four out of six amino acid residues that differ between UG2012 and NE2016 envelope proteins are located in the domain that contains the fusion loop (DII), we hypothesized that envelope protein mediates altered fusion kinetics. Using a time-to-fusion assay, we determined that NE2016 took longer to fuse than either UG2012 or NE2016^UG2012 E^. Since fusion rates are dependent on pH of the endosome and endosomal membrane composition (56), the difference in fusion rates between USUV viruses may be more profound in cells with slower endosomal maturation or altered membrane composition. Slower fusion may render a virus more susceptible to intracellular innate immune factors, particularly IFITM3, an interferon-inducible transmembrane protein that localizes to the membranes of late endosomes and can inhibit viral fusion of Influenza A virus, WNV, and dengue virus (57, 58). Additionally, *in vivo*, delayed fusion and lower initial viral output may limit NE2016’s ability to effectively escape the inoculation site and disseminate through the host before induction of innate immune responses, ultimately leading to reduced viral titers and less virulence. Future studies will address the mechanism of delayed fusion by NE2016 and its impact on cellular tropism and innate immune responses *in vitro* and *in vivo*.

We modeled the envelope protein structures of both UG2012ic and NE2016. The overall structures are highly similar, with the six amino acid differences having no discernible impact on the secondary or tertiary structures. A limitation of these structural studies is that we only modeled a single, static dimer of envelope protein. Like most flaviviruses, the USUV virion contains 180 copies of envelope protein that arrange themselves in 90 dimers in the prefusion form (47). During fusion, these dimers rearrange to form trimers (47). Since overall fusion kinetics differ between UG2012 and NE2016, we hypothesize that the conformation change from dimer to trimer occurs at a slower rate with NE2016 than with UG2012. Future structural studies will include dynamic modeling, which will allow us to ascertain whether the amino acid changes between NE2016 and UG2012 envelope proteins impact interactions between envelope dimers or the transition between the dimeric pre-fusion state and the trimeric fusion state. The two putative glycosylation sites in USUV envelope protein (E-154 and E-118; [47]) are not predicted to be impacted by the amino acid changes between NE2016 and UG2012. However, our structural analyses predicted additional glycosylation sites in both NE2016 (at E-88) and UG2012 (at E-52). These sites have yet to be validated experimentally. Modulation of envelope glycosylation sites has been shown to alter virulence in other flaviviruses, including Kunjin and Tembusu viruses (26, 59). Therefore, it would be pertinent to study envelope glycosylation in relation to USUV virulence.

 In this study, we focused on the 21 amino acid differences between NE2016 and UG2012; however, there are 268 nucleotide differences between the NE2016 and UG2012 genomes that do not result in amino acid changes: 1 in the 5′ untranslated region (UTR), 15 in the 3′UTR, and 252 silent mutations throughout the coding sequence. Because the secondary (hairpins, pseudoknots, etc.) and tertiary (long-range interactions) structures of flavivirus RNA are crucial for viral replication and antagonizing host immune responses, even silent mutations may impact flavivirus virulence (60, 61). Indeed, altering codon optimization of flavivirus genomes, which introduces numerous silent mutations, results in viral attenuation and is being explored as a method of creating vaccine strains of flaviviruses (61–63). Thus, it is possible that silent mutations between NE2016 and UG2012 could contribute to differential virulence. Future studies could address whether NE2016 and UG2012 differ in RNA structure and whether the UTRs impact differential virulence.

Phylogenetic analysis revealed that the six amino acids in the envelope protein of NE2016 that differ from UG2012 (E52, E88, E179, E231, E238, and E344) are consistently found in USUV strains from the Africa 3 lineage. USUV sequences from all other lineages (Africa 2, Europe 1, Europe 2, Europe 3, and Europe 5) matched UG2012 at these six residues in envelope. Here and previously, we have shown that NE2016 is less virulent in a mouse model of disease than UG2012 and other USUV isolates (Spain 2009, Senegal 2003, and South Africa 1959 [SAAR-1776]) (20). However, we have previously shown that NE2016 is not attenuated in two bird models—2-day-old chickens (*Gallus gallus*) and wild-caught house sparrows (*Passer domesticus*)—and in *Culex quinquefasciatus* mosquitoes (64, 65). Thus, while the amino acid changes in envelope between NE2016 and UG2012 may contribute to differential virulence in mammalian models, they appear to have little effect on the ability of NE2016 to replicate in bird models and mosquitoes. Humans and other mammals are dead-end hosts for USUV. Therefore, there is no evolutionary pressure for USUV to replicate efficiently or modulate virulence in mammals.

USUV is a flavivirus capable of causing severe disease in humans. The mechanisms through which USUV causes disease in humans are not understood. Through investigation of USUV strains that cause differing levels of virulence in a mouse model, we identified envelope as a viral factor that contributes to virulence and cell fusion. Future studies will investigate whether these specific components of USUV alter neuroinvasion. This information is key to (i) being able to predict the likelihood of a given USUV isolate to cause neuroinvasive disease and (ii) the development of specific anti-viral treatments to prevent or lessen USUV neuroinvasive disease. Furthermore, information gained from studying USUV may be applied to other closely related flaviviruses, like WNV or St. Louis encephalitis virus.

## Data Availability

All raw data used to create the figures in this study are available in Supplemental Materials.

## SUPPLEMENTAL MATERIAL

Supplemental text and figures mbio.01593-25-s0001.docx Additional experimental details; supplemental figures and tables.

Supplemental material mbio.01593-25-s0002.xlsx Data that were used to construct figures in main and supplemental text.

ASM does not own the copyrights to Supplemental Material that may be linked to, or accessed through, an article. The authors have granted ASM a non-exclusive, world-wide license to publish the Supplemental Material files. Please contact the corresponding author directly for reuse.
