# Supplementary material for: Naturally occurring mutations in envelope mediate virulence of Usutu virus
Source: mBio. 2025 Sep 12;16(10):e01593-25. doi: 10.1128/mbio.01593-25 (PMC12506040; doi:10.1128/mbio.01593-25)
Supplement: Supplemental text and figures — Additional experimental details; supplemental figures and tables. [file mbio.01593-25-s0001.docx]

**SUPPLEMENTAL METHODS**

**Generation of USUV infectious clones**

The infectious clones for UG2012 and NE2016 were re-cloned into an OriC plasmid to facilitate downstream cloning [1, 2]. Clones were designed *in silico* using SnapGene v.6.0.2 software (GSL Biotech). PCR primers were designed with 40bp overlaps. The NE2016 infectious clone without the OriC sequence was used as a template for PCR [1]. DNA fragments of UG2012 were synthesized (Twist Biosciences) and used as PCR templates. The PCR products were purified using the Machary-Nagel NucleoSpin Gel and PCR Clean-up kit (Item Number 740609). The amplicons were quantified by Qubit and mixed at an equimolar ratio. The amplicon mix was then assembled using the OriCiro 2x Recombination Assembly (RA) mix and incubated for 30 minutes at 42°C, followed by a 65°C step for 2 minutes. The assembled product was then amplified using the OriC amplification mix. The amplification mixture was primed by incubating at 33°C for 15 minutes. After priming, the assembly reaction was mixed into the amplification mix and incubated for 6 hours at 33°C and then held at 12°C. The next day, the product was supercoiled by diluting the product two-fold in 1x Amplification Buffer and incubating at 33 °C for 30 minutes. The supercoiled product was then tested by restriction digestion to confirm proper assembly. The remaining supercoiled product was diluted to a final concentration of 20 mM in EDTA.

**Generation of chimeric viruses**

Chimeric viruses were designed *in silico* using SnapGene (v.8.0.1) and the infectious clones as templates. Multigene chimeras were created by PCR amplifying four fragments from each infectious clone: (1) C-prM-E; (2) NS1-NS2a-NS2b; (3) NS3-NS4a-NS4b-NS5; and (4) untranslated regions (UTRs) and vector backbone. Appropriate fragments from UG2012 and NE2016 were combined to create multigene chimeras. Single gene chimeras used fragments 2-4 from the multigene chimeras and two additional fragments that were PCR amplified from each infectious clone: (1a) C-prM and (1b) E. Fragments were designed to have 40bp overlaps with each other. PCR products were then subjected to restriction endonuclease digestion to linearize and disrupt any residual infectious clone template. PCR products were purified via gel extraction using the Qiaquick gel extraction kit (Qiagen). Chimeras were assembled and initially amplified using the OriCiro kit following the manufacturer’s instructions. Additional amplification was performed via rolling circle amplification using Femtophi (Evomics) or Equiphi (Thermo) following manufacturer’s instructions. Following amplification, chimeras were diluted 1:20 in Jet Optimus buffer (Polyplus) and vortexed to disrupt concatemers.

**Generation of point mutants**

NE2016 envelope point mutants were designed in SnapGene (v.8.0.1). Six separate envelope fragments (one for each point mutant) were synthesized (Twist Biosciences). Fragments 1a, 2, 3, and 4 from single gene and multigene chimeras were used for assembly. Point mutants were assembled and amplified as described above.

**Rescue of infectious clones, chimeric viruses, and point mutants**

Viruses were rescued in Vero or BHK-21 cells seeded in 6-well plates. Once cells reached 60-80% confluency, they were transfected with 2ug of DNA per well using the Jet Optimus transfection reagent (Polyplus) following the manufacturer’s instructions. Cell culture media was changed 4 hours post transfection. Cells were monitored for cytopathic effect (CPE) daily. When greater than 50% of CPE was observed in wells, virus was harvested by transferring cell supernatant to a 50 mL conical tube and centrifuging at 500 x g for 10 minutes at 4^o^C to pellet cell debris. The supernatant was transferred to a new tube and centrifuged again before aliquoting and freezing at -80°C. Fresh cell culture media was added to cells to allow for an additional harvest the following day. Viral sequences were confirmed via amplicon-based Sanger sequencing or Nanopore sequencing (Plasmidsaurus). Sequence assembly and analysis was performed using Geneious Prime (v.2024.0.3).

***In vitro* growth curves**

Growth kinetics of all viruses were assessed in Vero cells seeded in 12-well plates. Once cells reached 80% confluency, they were inoculated with virus at a multiplicity of infection (MOI) of 0.1 or MOI of 10 and an inoculum volume of 150µL. Cells and virus mixture were incubated for 1 hour at 37^o^C. Then, cells were washed 3 times per well with 1mL phosphate buffered saline (PBS), and 1.5mL of cell culture media was added to each well. Sixty microliters of supernatants were collected daily for five days and stored at ‑80°C until titration via plaque assay. For growth curves conducted at an MOI of 10, supernatants were collected at 12 hours post-inoculation.

**Plaque assay**

Plaque assays were performed to titrate samples. Ten-fold serial dilutions of samples were performed in BA-1 media. Brain samples were homogenized prior to serial dilution. To each brain, two times the volume (in mL) of BA-1 per brain mass (in g) and a 3mm metal bead were added. Samples were homogenized using a TissueLyzer (Qiagen) at 20 oscillations/s for 3 minutes and remaining solids were pelleted by centrifugation at 10,000 x g for 2 minutes. Virus dilutions were added to 12-well plates containing Vero cells at 100% confluency. Virus and cells were incubated for 1 hour at 37^o^C in a humidified incubator after which an agarose overlay containing Ye-Lah media was added and allowed to solidify. Cells were incubated for 2 days, and then, a second overlay containing neutral red was added. After an additional day of incubation, plaques were counted. The limit of detection was determined by calculating the titer of a hypothetical sample that had 1 plaque in the lowest dilution. Samples that had no detectable virus were plotted at half the limit of detection to distinguish them from samples that had detectable, but low, viral titers.

**Histological analysis**

Formalin fixed brain samples were paraffin-embedded, and 5μm slices were attached to charged glass slides. Slides were stained with Hematoxylin and Eosin (H&E) following normal procedures. Slides from uninfected mice served as controls. Slides were analyzed by Sheryl Coutermarsh-Ott, DVM, PhD, Diplomate of the American College of Veterinary Pathologists (DACVP), who was blinded throughout the analysis. Parameters assessed included parenchymal inflammation, perivascular inflammation, cell necrosis, and leptomeningitis. The parameters were chosen to identify microscopic features of tissue damage, with special consideration for those typically observed in viral infection. Parameters were subjectively scored as 0 (no lesions present), 1 (mild lesions), 2 (moderate lesions), or 3 (severe lesions). Once individual scores were assigned, a total histopathologic score for each sample was calculated by summing the individual parameter scores.

**Structure modelling methods**

Structural models of USUV envelope proteins of NE2016 and UG2012 were generated using Robetta Comparative Modeling [3]. The crystal structure of USUV envelope protein in the pre-fusion state (PDB ID: 6A0P) was utilized in comparative modeling to increase structural accuracy [4, 5]. UG2012 and NE2016 models both passed the Verify3D structural analysis, had 0.0% Ramachandran outliers, and a QMEAN Z-score of 0.86; all indicative of confidence in each model [6-8]. PyMol (v 2.0) was used to visualize the prepared models [9]. NetNGlyc was used to identify glycosylation sites on both the Uganda and Netherlands strains and the likelihood of glycosylation [10]. The option to check every possible asparagine was enabled.

**Time-to-fusion assay**

Vero cells were seeded in 6-well plates. When cells reached approximately 90% confluency, wells were infected on ice with 1000 PFUs of virus. This virus concentration was chosen because it yielded between 50-100 plaques in the positive control wells of the completed assay. Inoculated plates were incubated at 4°C, rocking every 10 minutes, for one hour to allow virus to adsorb to cells. Afterwards, wells were each washed on ice twice with 1 mL of ice-cold PBS to remove unbound virus. Then, 500µL of buffered BA-1 (BA-1 containing 25mM HEPES buffer) was added to each well. For timepoint 0 wells, buffered BA-1 was immediately removed and replaced with 500µL of 200mM ammonium chloride (NH_4_Cl) solution (diluted in buffered BA-1). All plates were then incubated for 1 hour at 37°C. Every 15 minutes during incubation, buffered BA-1 was removed from another set of wells and replaced with an equal volume of NH_4_Cl. Wells that received no NH_4_Cl served as no treatment controls. Following a total incubation of 1 hour, wells were each washed twice with 1mL of PBS. Then, an agarose overlay containing Ye-Lah media was added to each well and allowed to solidify. Three days later, plates were fixed with 10% neutral buffered formalin, agarose plugs were removed, and plates were stained with 0.1% crystal violet solution. Plaques were counted in each well.

**Plaque reduction neutralization titer (PRNT) assay**

Neutralizing antibody titers in mouse serum were quantified using a PRNT assay as described in [11]. Briefly, serum collected from infected mice upon euthanasia was diluted 1:5 in sterile PBS and heat inactivated for 30 minutes at 56°C. Two-fold serial dilution series were made by diluting heat inactivated serum in media. Then, all dilutions were diluted 1:2 in USUV that had been prediluted to 1000 PFU/mL. USUV added to each dilution series of serum was the same virus with which the mice were originally infected (i.e. NE2016ic or NE2016^UG2012 E^). Virus diluted 1:2 in media served as a no-antibody control. Virus and antibody were incubated at 37°C for 1 hour. Then, virus/antibody mixtures were added to 6‑well plates containing Vero cells at 100% confluency. From here on, plates were treated as in plaque assay. To calculate PRNT_50_ titer, percent inhibition was graphed as a function of the reciprocal serum titer (log_10_ transformed) in GraphPad Prism. Non-linear regression was performed using the sigmoidal dose response (variable slope) equation. PRNT_50_ value was interpolated based on the results of the regression.

**SUPPLEMENTAL FIGURES**

**Figure S1: Netherlands 2016 (NE2016) infectious clone (ic), chimeras, and point mutants have similar growth kinetics in Vero cells.**

Growth kinetics of (**A**) Uganda 2012 (UG2012)ic and NE2016ic, (**B**) NE2016 multigene chimeras, (**C**) NE2016 single gene chimeras, and (**D**) NE2016 envelope point mutants in Vero cells after infection at MOI of 0.1. Data were analyzed via repeated measures ANOVA followed by multiple comparisons t-tests using Dunnett’s adjustment.

* p<0.05; ***p<0.001


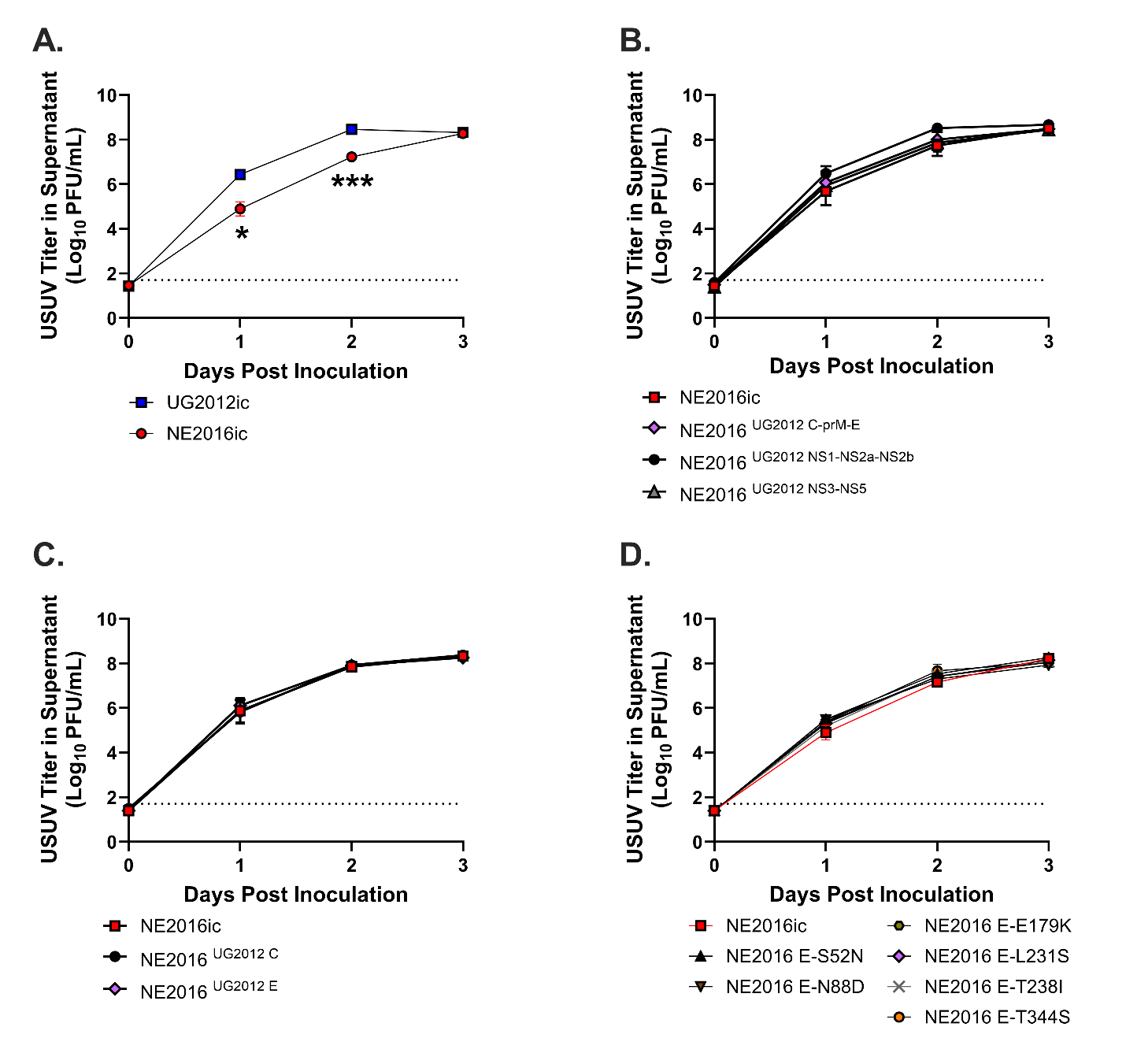


**Figure S2: Individual weight curves of mice infected with NE2016ic or UG2012ic**

Weight curves of individual mice infected with either NE2016ic (**A**) or UG2012ic (**B**) from the study shown in **Figure 1**. Each line and set of points represent a separate mouse. White symbols represent timepoints where a planned euthanasia occurred (as opposed to a humane endpoint).


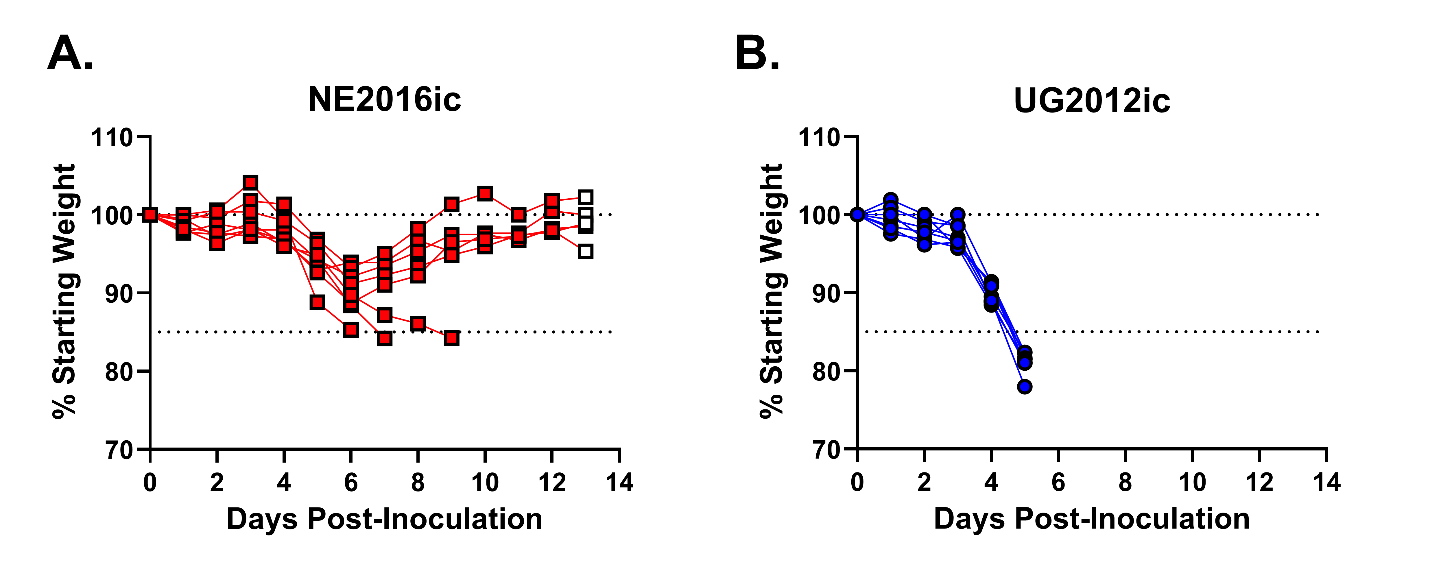


**Figure S3: Individual weight curves of mice infected with NE2016ic or multigene chimeras**

Weight curves of individual mice infected with NE2016ic (**A**), NE2016^UG2012 C-prM-E^ (**B**), NE2016^UG2012 NS1-NS2a-NS2b^ (**C**), or NE2016^UG2012 NS3-NS5^ (**D**) from the study shown in **Figure 2**. Each line and set of points represent a separate mouse. White symbols represent timepoints where a planned euthanasia occurred (as opposed to a humane endpoint).


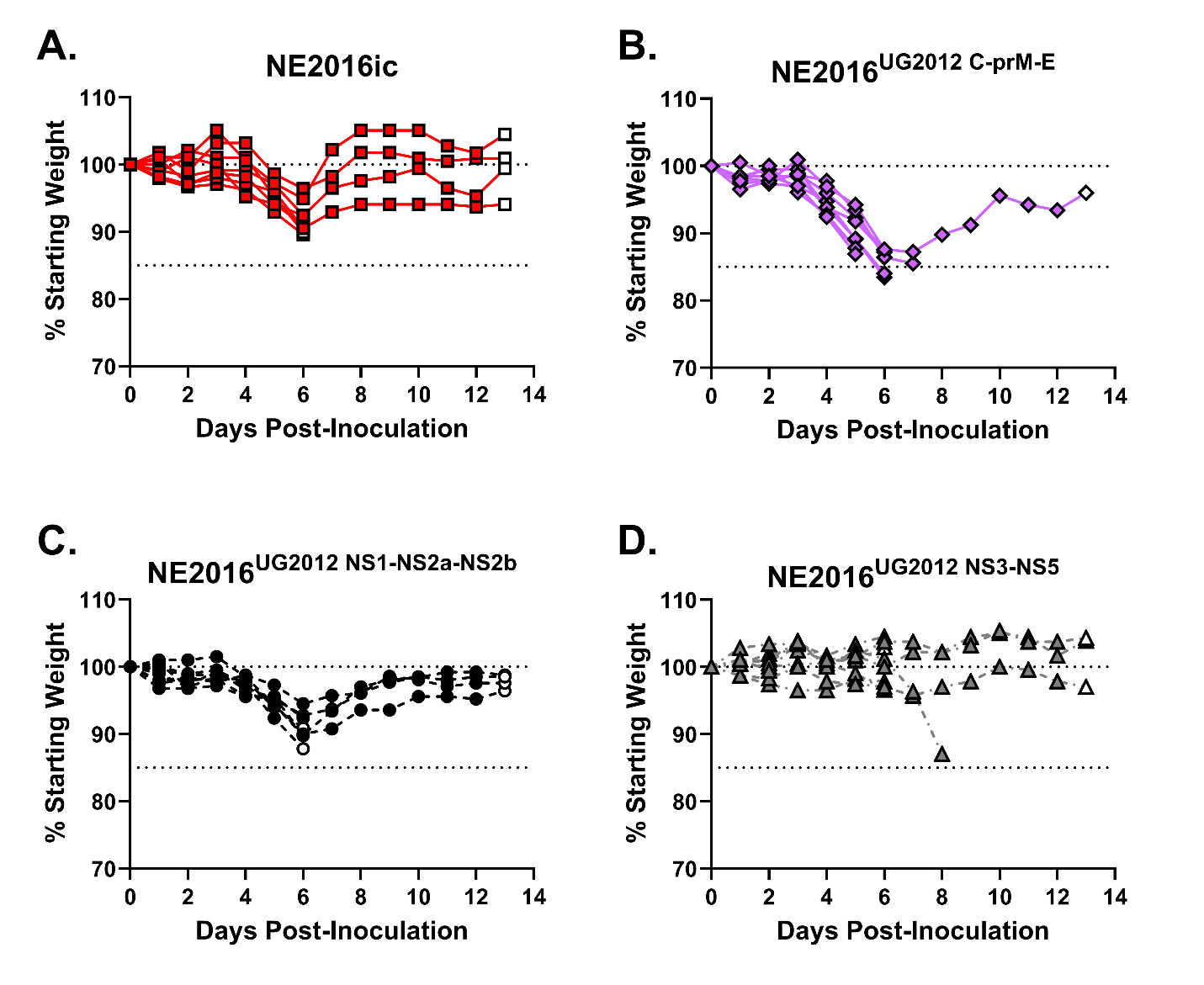


**Figure S4: Individual weight curves of mice infected with NE2016ic or single gene chimeras**

Weight curves of individual mice infected with NE2016ic (**A**), NE2016^UG2012 C^ (**B**), or NE2016^UG2012 E^ (**C**) from the study shown in **Figure 3**. Each line and set of points represent a separate mouse. White symbols represent timepoints where a planned euthanasia occurred (as opposed to a humane endpoint).


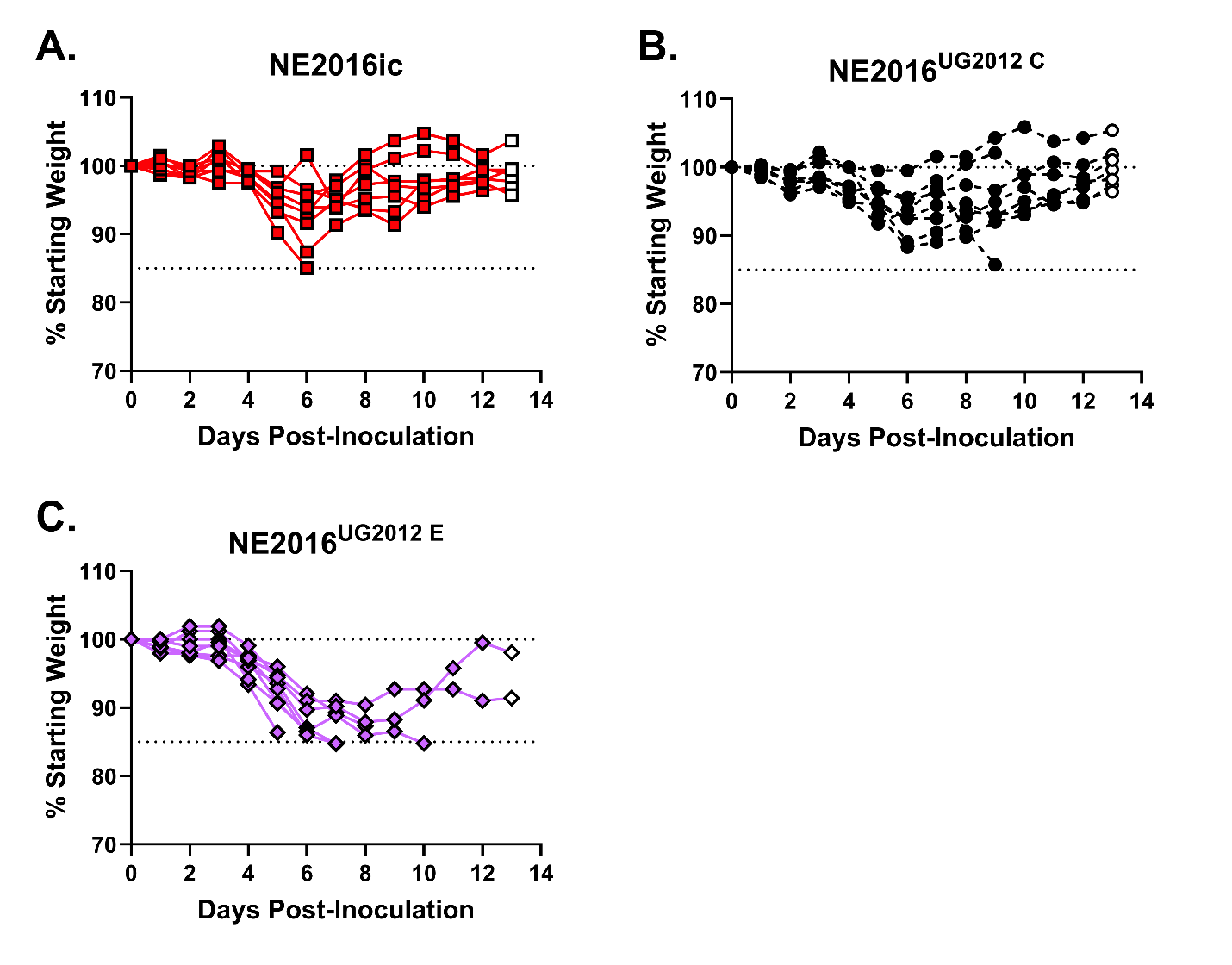


**Figure S5: Individual weight curves of mice infected with NE2016ic or point mutants**

Weight curves of individual mice infected with NE2016ic (**A**), NE2016 E-S52N (**B**), NE2016 E-N88D (**C**), NE2016 E-E179K (**D**), NE2016 E-L231S (**E**), NE2016 E-T238I (**F**), NE2016 E-T344S (**G**) from the study shown in **Figure 4**. Each line and set of points represent a separate mouse. White symbols represent timepoints where a planned euthanasia occurred (as opposed to a humane endpoint).


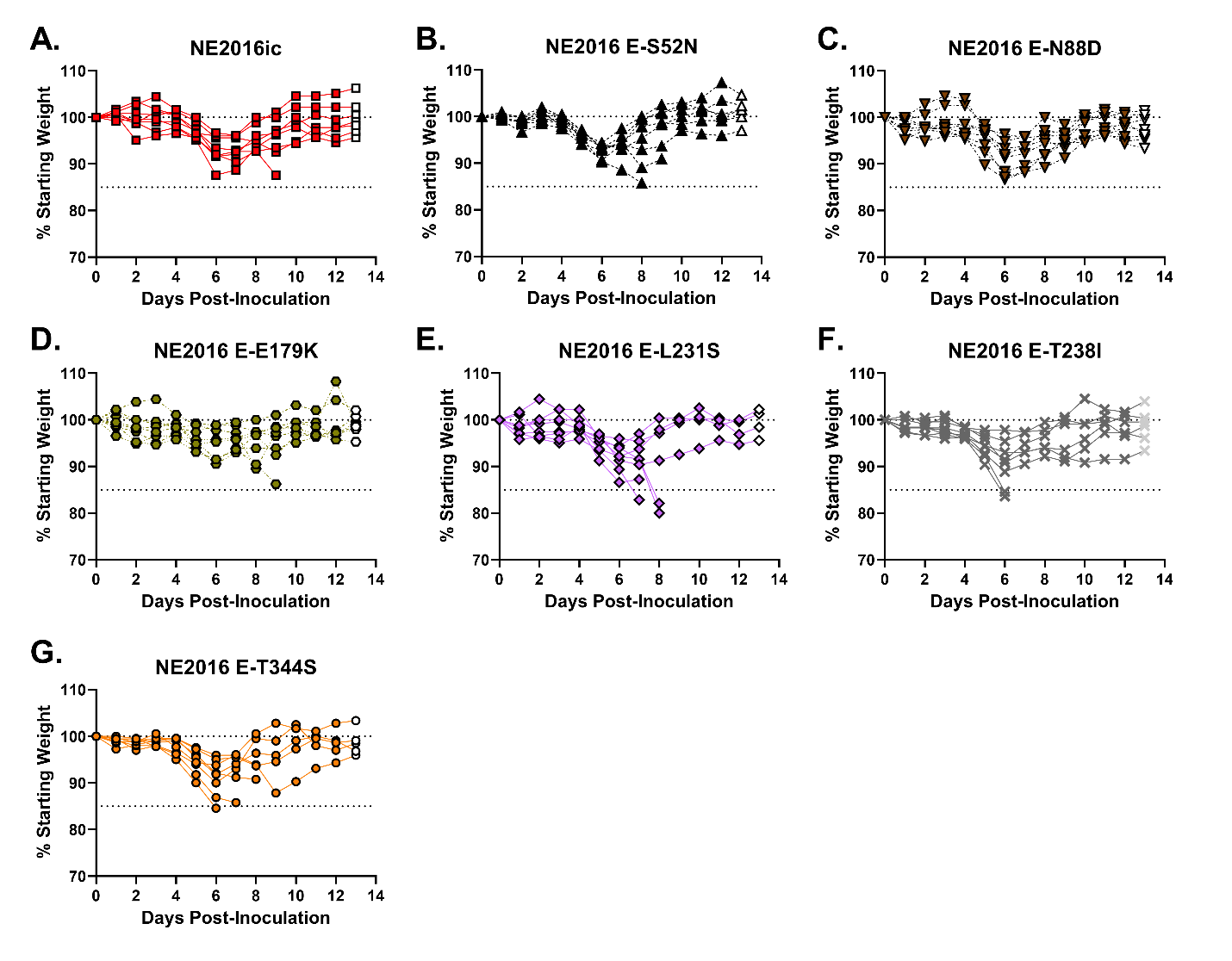


**Figure S6: Additional biological replicates of single step growth curve**

Single step growth curves were performed in Vero cells **(A-C).** Cells were infected at MOI of 10 with NE2016ic (red squares), UG2012ic (blue circles), or NE2016^UG2012 E^ (lavender diamonds). Data are the mean and standard deviation of 3 technical replicates of single biological replicates. (One biological replicate per plot). Data were analyzed via 2-way ANOVA with multiple comparisons t-test using the Dunnett’s correction. Asterisks are colored to show which virus was significantly different from NE2016ic.

**
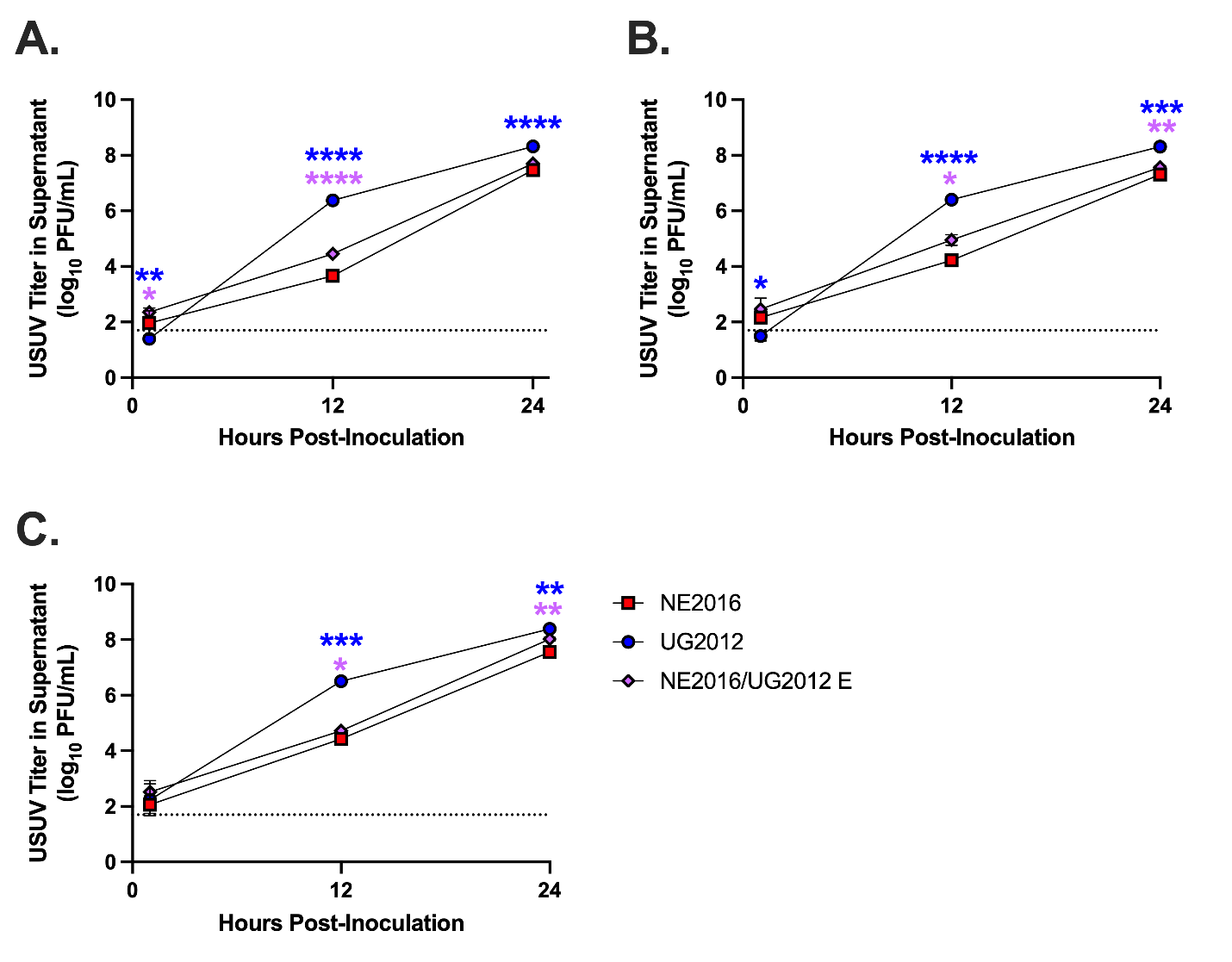
**

**Table S1: Mice that were moribund at time of euthanasia**

All groups started with 8 mice. Mice could have met either or both weight and moribund criteria for euthanasia.

| **Figure** | **Group** | **Total mice euthanized early** | **Mice euthanized due to weight loss** | **Mice that were moribund** |
| --- | --- | --- | --- | --- |
| 1 | NE2016ic | 3 | 3 | 0 |
| 1 | UG2012ic | 8 | 8 | 6 |
| 2 | NE2016ic | 0 | 0 | 0 |
| 2 | NE2016^UG2012 C-prM-E^ | 5 | 5 | n.d. |
| 2 | NE2016^UG2012 NS1-NS2a-NS2b^ | 0 | 0 | 0 |
| 2 | NE2016^UG2012 NS3-NS5^ | 1 | 1 | n.d. |
| 3 | NE2016ic | 1 | 1 | 0 |
| 3 | NE2016^UG2012 C^ | 1 | 1 | 0 |
| 3 | NE2016^UG2012 E^ | 6 | 6 | 0 |
| 4 | NE2016ic | 1 | 1 | 0 |
| 4 | NE2016 E-S52N | 2 | 1 | 1 |
| 4 | NE2016 E-N88D | 0 | 0 | 0 |
| 4 | NE2016 E-E179K | 1 | 1 | 0 |
| 4 | NE2016 E-L231S | 4 | 4 | 2 |
| 4 | NE2016 E-T238I | 2 | 2 | 0 |
| 4 | NE2016 E-T344S | 3 | 2 | 1 |

n.d. = no data

**Table S2: USUV sequences used in phylogenetic analysis**

Residues in light blue match UG2012, and residues in light red match NE2016

|  |  | Amino Acid at envelope residue: | | | | | |
| --- | --- | --- | --- | --- | --- | --- | --- |
| Accession No. (GenBank) | Lineage | E-52 | E-88 | E-179 | E-231 | E-238 | E-344 |
| KC754954 | Africa 2 | N | D | K | S | I | S |
| KF573410 | Africa 2 | N | D | K | S | I | S |
| KX601692 | Africa 2 | N | D | K | S | I | S |
| MH727240 | Africa 2 | N | D | K | S | I | S |
| MH727242 | Africa 2 | N | D | K | S | I | S |
| MN813488 | Africa 2 | N | D | K | S | I | S |
| MN813489 | Africa 2 | N | D | K | S | I | S |
| MN813492 | Africa 2 | N | D | K | S | I | S |
| ON813229 | Africa 2 | N | D | K | S | I | S |
| KC754955 | Africa 3 | S | N | E | L | T | T |
| KM659877 | Africa 3 | S | N | E | L | T | T |
| KY128482 | Africa 3 | S | N | E | L | T | T |
| KY263625 | Africa 3 | S | N | E | L | T | T |
| LR989894 | Africa 3 | S | N | E | L | T | T |
| MH891847 | Africa 3 | S | N | E | L | T | T |
| MK230891 | Africa 3 | S | N | E | L | T | T |
| MN122256 | Africa 3 | S | N | E | L | T | T |
| MN813490  (NE2016) | Africa 3 | S | N | E | L | T | T |
| MT795154 | Africa 3 | S | N | E | L | T | T |
| MW001216 | Africa 3 | S | N | E | L | T | T |
| OM202464 | Africa 3 | S | N | E | L | T | T |
| ON755222 | Africa 3 | S | N | E | L | T | T |
| OP007489 | Africa 3 | S | N | E | L | T | T |
| OP422562 | Africa 3 | S | N | E | L | T | T |
| OP422565 | Africa 3 | S | N | E | L | T | T |
| OP921076 | Africa 3 | S | N | E | L | T | T |
| OQ630905 | Africa 3 | S | N | E | L | T | T |
| OQ630906 | Africa 3 | S | N | E | L | T | T |
| OQ630908 | Africa 3 | S | N | E | L | T | T |
| OR141586 | Africa 3 | S | N | E | L | T | T |
| KY294723 | Africa 3 | S | N | E | S | T | T |
| MK419834 | Africa 3 | S | N | E | S | T | T |
| MT863562 | Africa 3 | S | N | E | S | T | T |
| OU674388 | Africa 3 | S | N | E | S | T | T |
| AY453411 | Europe 1 | N | D | K | S | I | S |
| MG888044 | Europe 1 | N | D | K | S | I | S |
| JF266698 | Europe 2 | N | D | K | S | I | S |
| KU573074 | Europe 2 | N | D | K | S | I | S |
| KU573076 | Europe 2 | N | D | K | S | I | S |
| KX268471 | Europe 2 | N | D | K | S | I | S |
| KX555624 | Europe 2 | N | D | K | S | I | S |
| MF063042 | Europe 2 | N | D | K | S | I | S |
| MF063043 | Europe 2 | N | D | K | S | I | S |
| MF991886 | Europe 2 | N | D | K | S | I | S |
| MN989418 | Europe 2 | N | D | K | S | I | S |
| MT784899 | Europe 2 | N | D | K | S | I | S |
| MW164657 | Europe 2 | N | D | K | S | I | S |
| MW164769 | Europe 2 | N | D | K | S | I | S |
| MW164770 | Europe 2 | N | D | K | S | I | S |
| MW164771 | Europe 2 | N | D | K | S | I | S |
| ON032487 | Europe 2 | N | D | K | S | I | S |
| ON813235 | Europe 2 | N | D | K | S | I | S |
| ON813236 | Europe 2 | N | D | K | S | I | S |
| OP422564 | Europe 2 | N | D | K | S | I | S |
| OP734261 | Europe 2 | N | D | K | S | I | S |
| OR141598 | Europe 2 | N | D | K | S | I | S |
| OR757497 | Europe 2 | N | D | K | S | I | S |
| OR921106 | Europe 2 | N | D | K | S | I | S |
| PP104391 | Europe 2 | N | D | K | S | I | S |
| KX601691 | Europe 3 | N | D | K | S | I | S |
| KY426770 | Europe 3 | N | D | K | S | I | S |
| MK230892 | Europe 3 | N | D | K | S | I | S |
| MT133690 | Europe 3 | N | D | K | S | I | S |
| MT580899 | Europe 3 | N | D | K | S | I | S |
| OP422563 | Europe 3 | N | D | K | S | I | S |
| OP555958 | Europe 3 | N | D | K | S | I | S |
| OQ630904 | Europe 3 | N | D | K | S | I | S |
| OR141586 | Europe 3 | N | D | K | S | I | S |
| OR573992 | Europe 3 | N | D | K | S | I | S |
| OR573993 | Europe 3 | N | D | K | S | I | S |
| MG461313 | Europe 5 | N | D | K | S | I | S |
| MN813491  (UG2012) | Europe 5 | N | D | K | S | I | S |
| ON813224 | Europe 5 | N | D | K | S | I | S |
| ON813225 | Europe 5 | N | D | K | S | I | S |

**SUPPLEMENTAL REFERENCES**

1. Bates TA, Chuong C, Hawks SA, Rai P, Duggal NK, Weger-Lucarelli J. Development and characterization of infectious clones of two strains of Usutu virus. Virology. 2021;554:28-36. Epub 2020/12/23. doi: 10.1016/j.virol.2020.12.004. PubMed PMID: 33352463.

2. Marano JM, Cereghino C, Finkielstein CV, Weger-Lucarelli J. An in vitro workflow to create and modify infectious clones using replication cycle reaction. Virology. 2023;585:109-16. Epub 20230613. doi: 10.1016/j.virol.2023.05.013. PubMed PMID: 37331111; PubMed Central PMCID: PMCPMC10528026.

3. Baek M, DiMaio F, Anishchenko I, Dauparas J, Ovchinnikov S, Lee GR, et al. Accurate prediction of protein structures and interactions using a three-track neural network. Science. 2021;373(6557):871-6. Epub 2021/07/21. doi: 10.1126/science.abj8754. PubMed PMID: 34282049; PubMed Central PMCID: PMCPMC7612213.

4. Berman HM, Westbrook J, Feng Z, Gilliland G, Bhat TN, Weissig H, et al. The Protein Data Bank. Nucleic Acids Res. 2000;28(1):235-42. Epub 1999/12/11. doi: 10.1093/nar/28.1.235. PubMed PMID: 10592235; PubMed Central PMCID: PMCPMC102472.

5. Chen Z, Ye F, Lin S, Yang F, Cheng Y, Cao Y, et al. Crystal structure of Usutu virus envelope protein in the pre-fusion state. Virol J. 2018;15(1):183. Epub 2018/11/28. doi: 10.1186/s12985-018-1092-6. PubMed PMID: 30477514; PubMed Central PMCID: PMCPMC6260896.

6. Luthy R, Bowie JU, Eisenberg D. Assessment of protein models with three-dimensional profiles. Nature. 1992;356(6364):83-5. Epub 1992/03/05. doi: 10.1038/356083a0. PubMed PMID: 1538787.

7. Bowie JU, Luthy R, Eisenberg D. A method to identify protein sequences that fold into a known three-dimensional structure. Science. 1991;253(5016):164-70. Epub 1991/07/12. doi: 10.1126/science.1853201. PubMed PMID: 1853201.

8. Waterhouse A, Bertoni M, Bienert S, Studer G, Tauriello G, Gumienny R, et al. SWISS-MODEL: homology modelling of protein structures and complexes. Nucleic Acids Res. 2018;46(W1):W296-W303. Epub 2018/05/23. doi: 10.1093/nar/gky427. PubMed PMID: 29788355; PubMed Central PMCID: PMCPMC6030848.

9. Schrodinger L. The PyMOL Molecular Graphics System, Version 2.0. 2020.

10. Gupta R, Brunak S. Prediction of glycosylation across the human proteome and the correlation to protein function. Pac Symp Biocomput. 2002:310-22. Epub 2002/04/04. PubMed PMID: 11928486.

11. Salgado R, Hawks SA, Frere F, Vazquez A, Huang CY, Duggal NK. West Nile Virus Vaccination Protects against Usutu Virus Disease in Mice. Viruses. 2021;13(12). Epub 2021/12/29. doi: 10.3390/v13122352. PubMed PMID: 34960621; PubMed Central PMCID: PMCPMC8704473.
